# Gene domain-specific DNA methylation episignatures highlight distinct molecular entities of ADNP syndrome

**DOI:** 10.1186/s13148-019-0658-5

**Published:** 2019-04-27

**Authors:** Eric G. Bend, Erfan Aref-Eshghi, David B. Everman, R. Curtis Rogers, Sara S. Cathey, Eloise J. Prijoles, Michael J. Lyons, Heather Davis, Katie Clarkson, Karen W. Gripp, Dong Li, Elizabeth Bhoj, Elaine Zackai, Paul Mark, Hakon Hakonarson, Laurie A. Demmer, Michael A. Levy, Jennifer Kerkhof, Alan Stuart, David Rodenhiser, Michael J. Friez, Roger E. Stevenson, Charles E. Schwartz, Bekim Sadikovic

**Affiliations:** 1 Greenwood Genetic Center, 106 Gregor Mendel Cir, Greenwood, SC 29646 USA; 2 PreventionGenetics, Marshfield, WI USA; 3 Department of Pathology and Laboratory Medicine, Western University, 800 Commissioner’s Road E, London, ON N6A 5W9 Canada; 4 Molecular Genetics Laboratory, Victoria Hospital, London Health Sciences Centre, London, ON Canada; 5 Al DuPont Hospital for Children, Wilmington, DE USA; 6 Center for Applied Genomics, Children’s Hospital of Philadelphia, Philadelphia, PA USA; 7 Division of Human Genetics, Children’s Hospital of Philadelphia, Philadelphia, PA USA; 8 Spectrum Health, Grand Rapids, MI USA; 9 Levine Children’s Hospital, Carolinas Medical Center, Charlotte, NC USA; 10 Department of Pediatrics, Biochemistry and Oncology, Western University, London, ON Canada

**Keywords:** Epigenetics, Episignature, DNA methylation, ADNP, Helsmoortel-Van der Aa syndrome, Autism, Intellectual disability, Unresolved clinical cases, Disease screening

## Abstract

**Background:**

ADNP syndrome is a rare Mendelian disorder characterized by global developmental delay, intellectual disability, and autism. It is caused by truncating mutations in *ADNP*, which is involved in chromatin regulation. We hypothesized that the disruption of chromatin regulation might result in specific DNA methylation patterns that could be used in the molecular diagnosis of ADNP syndrome.

**Results:**

We identified two distinct and partially opposing genomic DNA methylation episignatures in the peripheral blood samples from 22 patients with ADNP syndrome. The “epi-ADNP-1” episignature included ~ 6000 mostly hypomethylated CpGs, and the “epi-ADNP-2” episignature included ~ 1000 predominantly hypermethylated CpGs. The two signatures correlated with the locations of the *ADNP* mutations. Epi-ADNP-1 mutations occupy the N- and C-terminus, and epi-ADNP-2 mutations are centered on the nuclear localization signal. The episignatures were enriched for genes involved in neuronal system development and function. A classifier trained on these profiles yielded full sensitivity and specificity in detecting patients with either of the two episignatures. Applying this model to seven patients with uncertain clinical diagnosis enabled reclassification of genetic variants of uncertain significance and assigned new diagnosis when the primary clinical suspicion was not correct. When applied to a large cohort of unresolved patients with developmental delay (*N* = 1150), the model predicted three additional previously undiagnosed patients to have ADNP syndrome. DNA sequencing of these subjects, wherever available, identified pathogenic mutations within the gene domains predicted by the model.

**Conclusions:**

We describe the first Mendelian condition with two distinct episignatures caused by mutations in a single gene. These highly sensitive and specific DNA methylation episignatures enable diagnosis, screening, and genetic variant classifications in ADNP syndrome.

**Electronic supplementary material:**

The online version of this article (10.1186/s13148-019-0658-5) contains supplementary material, which is available to authorized users.

## Background

ADNP syndrome (Helsmoortel-van der Aa syndrome; OMIM# 615873) is caused by dominant negative truncating variants in *ADNP* [1]. This disorder is among the most common causes of syndromic autism spectrum disorder (ASD) and intellectual disability (ID) and frequently tops the list of genes mutated in large-scale neurodevelopmental disorder sequencing cohorts [2, 3]. As a group, neurodevelopmental disorders are difficult to diagnose clinically, and ADNP syndrome exemplifies the challenges faced in this area. The major clinical features of ADNP syndrome include global developmental delay, ID, and ASD. These are often accompanied by additional comorbid features with variable expressivity (i.e., hypotonia, gastrointestinal, behavioral and sleep problems) [4]. In order to advance our ability to diagnose and eventually treat ADNP syndrome and similar conditions, additional biomarkers will be useful for variant classification, cohort screening, and functional analysis. Whole genome methylation analysis offers significant promise to realize these goals.

The *ADNP* gene encodes activity-dependent neuroprotective protein (ADNP), which is ubiquitously expressed and involved in chromatin remodeling and gene expression. ADNP is important for brain development and may be linked to cognitive ability [5, 6]. The sequence motifs of the ADNP protein include nine zinc-fingers, a nuclear localization signal, a DNA-binding homeobox motif, and an HP1-binding motif. In most cell types, ADNP is localized in the nucleus and recruited to histone H3K9me3 marked heterochromatin by HP1 [7]. ADNP directly interacts with members of the BRG1/Brm-associated factor (BAF) complex including ARID1A, SMARCA4, and SMARCC2 [1, 8]. The BAF complex (SWI/SNF in yeast and Brahma in Drosophila) is a 15-subunit protein complex that modifies the placement of nucleosomes along the length of DNA molecules by hydrolyzing ATP. The BAF family of chromatin remodelers regulates gene expression, thereby influencing cell differentiation, neural development, and learning and memory [9].

The identification of human neurodevelopmental disorders caused by genes involved in chromatin regulation is increasing and now includes more than 28 genes encoding chromatin regulators [10]. Eight of the 15 subunits of the BAF complex have been implicated in neurodevelopmental disorders [10]. Pathogenic variants in these genes cause Coffin-Siris syndrome and Nicolaides-Baraitser syndrome; both have considerable phenotypic overlap with ADNP syndrome including global developmental delay, hypotonia, intellectual disability, gastrointestinal complications, and behavioral problems. Recently, defects in BAF complex members and several other chromatin remodeling genes have been shown to have syndrome-specific genome-wide DNA methylation signatures, the so-called episignatures [11–17]. These studies have shown that changing histone marks leads to alternative methylation of genomic DNA, and this phenomenon can be leveraged to diagnose disease.

Here, we demonstrate that ADNP syndrome co-occurs with unique genomic DNA methylation changes in the peripheral blood. Uniquely, defects in *ADNP* produce two episignatures with partially contrasting methylation patterns which correlate with the location of the mutations. We describe in details the overlap and dissimilarities of the two episignatures and demonstrate the enrichment of the harboring genes in neuronal system pathways. We show that these changes are specific to ADNP syndrome and do not occur in other neurodevelopmental conditions. By computational modeling of the two episignatures, we show that they can be successfully applied to resolve ambiguous clinical/molecular cases, provide new diagnosis when the initial clinical assumption is not correct, and identify novel ADNP cases through screening of a large cohort of undiagnosed subjects presenting with intellectual disability.

## Results

### Clinical description of patients with ADNP syndrome

This study included 22 subjects with confirmed clinical and molecular diagnoses of ADNP syndrome (Table 1). Nine of the individuals provided detailed clinical phenotype information, which was consistent with that reported in other studies [1, 4]. Briefly, intellectual disability, developmental delay, hypotonia, and ASD were the only consistent features. Comorbidities were diverse and sporadic. Early developmental delays were mild, and the average age at diagnosis was 5 years (range 1 year 3 months to 11 years 8 months). Facial dysmorphism is subtle in ADNP syndrome, and no consistent patterns were appreciated in our cohort. All 22 patients with definitive ADNP syndrome were diagnosed by gene panels or whole exome sequencing. All had premature termination variants, and were determined to be de novo by Sanger sequencing of the (self-reporting) parents or trio exome analysis. The most common *ADNP* nonsense variant described (p.Tyr719*) occurred in six patients resulting from c.2157C>A or c.2157C>G. Phenotypic features observed in patients with this mutation were representative of the entire cohort. This study includes the first patient reported with a truncating N-terminal mutation in the first coding exon (exon 3; c.103dupA; p.Ile35Asnfs*5; phenotype described in Additional file 1: Figure S1). This novel mutation broadens the mutational spectrum of ADNP syndrome.

**Table 1:** Twenty-two subjects with a confirmed clinical/molecular diagnosis of ADNP syndrome

ID	Age at blood draw	Sex	*ADNP* variant	Variant effect	Subtype	Dataset
ADNP_03^a^	11	M	c.103dupA (p.Ile35Asnfs*5)	Frame-shift	ADNP-1	Training
ADNP_17	12	F	c.190dupA (p.Thr64Asnfs*35)	Frame-shift	ADNP-1	Training
ADNP_16	4	M	c.539_542delTTAG (p.Val180Glyfs*17)	Frame-shift	ADNP-1	Testing
ADNP_25	5	M	c.819delC (p.Lys274Asnfs*31)	Frame-shift	ADNP-1	Training
ADNP_29	3	M	c.859_862dup (p.Gly288Aspfs*27)	Frame-shift	ADNP-1	Training
ADNP_23	10	M	c.1046_1047delTG (p.Leu349Argfs*49)	Frame-shift	ADNP-1	Testing
ADNP_08	2	M	c.1102C>T (p.Gln368*)	Nonsense	ADNP-1	Training
ADNP_05^a^	2	M	c.1106_1108delTACinsCTGT (p.Leu369Serfs*30)	Frame-shift	ADNP-1	Training
ADNP_12	4	F	c.1222_1223delAA (p.Lys408Valfs*31)	Frame-shift	ADNP-1	Training
ADNP_20	9	F	c.1287dupT (p.Ala430Cysfs*10)	Frame-shift	ADNP-1	Training
ADNP_15	12	F	c.2156_2157insA (p.Tyr719*)	Nonsense	ADNP-2	Training
ADNP_07	5	F	c.2157C>A (p.Tyr719*)	Nonsense	ADNP-2	Testing
ADNP_10	5	F	c.2157C>A (p.Tyr719*)	Nonsense	ADNP-2	Training
ADNP_14	5	M	c.2157C>A (p.Tyr719*)	Nonsense	ADNP-2	Training
ADNP_04	12	F	c.2157C>G (p.Tyr719*)	Nonsense	ADNP-2	Training
ADNP_11	4	F	c.2157C>G (p.Tyr719*)	Nonsense	ADNP-2	Testing
ADNP_21	4	M	c.2188C>T (p.Arg730*)	Nonsense	ADNP-2	Testing
ADNP_13	3	M	c.2268dup (p.Lys757Glnfs*4)	Frame-shift	ADNP-2	Training
ADNP_24	10	M	c.2287delT (p.Ser763Profs*9)	Frame-shift	ADNP-2	Training
ADNP_22	3	M	c.2287dupT (p.Ser763Phefs*3)	Frame-shift	ADNP-2	Training
ADNP_02^a^	8	M	c.2340T>G (p.Tyr780*)	Nonsense	ADNP-2	Training
ADNP_09	12	M	c.2419_2423delAAAAG (p.Lys807Glufs*6)	Frame-shift	ADNP-1	Testing

^a^The DNA methylation profiles of these three subjects were examined from blood samples collected years apart to evaluate the changes in the ADNP episignature over time (Additional file 1: Figure S1). Individuals are listed in ascending order according to the cDNA nomenclature. Mean ± standard deviation of all patients’ age 6.8 ± 3.8 (36% females), matched controls 7.9 ± 5.7 (37% females); epi-ADNP-1 subtype 7.2 ± 4.2 (25% females), matched controls 7.3 ± 4.2 (28% females); epi-ADNP-2 subtype age 6.3 ± 3.6 (32% females), matched controls 5.6 ± 3.6 (35% females); ADNP transcript NM_015339.2; Photographs of some of these subjects are provided in Additional file 1: Figure S1

### Mutations in *ADNP* cause two distinct episignatures with partially opposite DNA methylation profiles

**Fig. 1:**
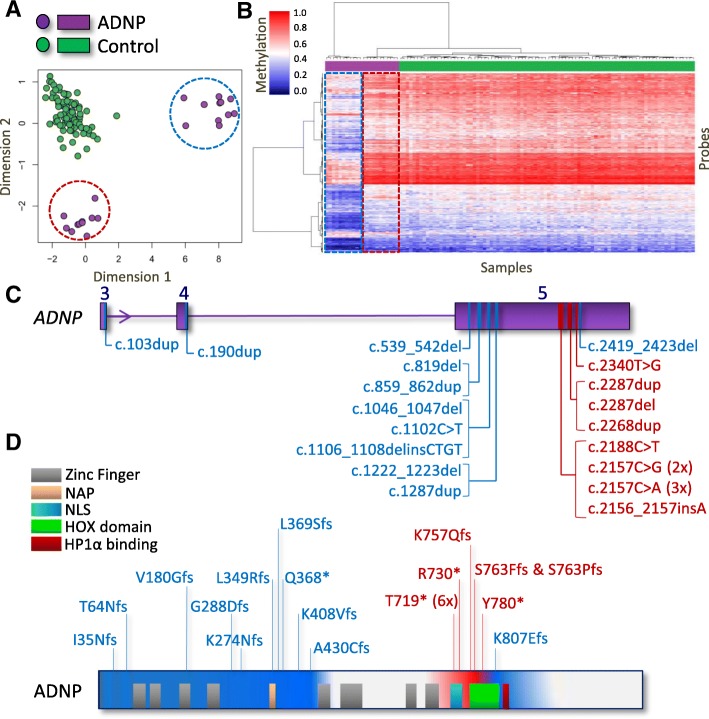
Correlation between the genetic coordinates of the *ADNP* mutations and two ADNP episignatures. Comparison of patients with ADNP syndrome and controls identified 1320 differentially methylated CpG sites. **a** Illustration of the top two dimensions of the multiple dimensional scaling of the patients (purple) and controls (green) using these probes reveals that while patients are separated from controls, they are clustered in two groups (indicated with dashed circles) with greater distances from each other than from controls. **b** A hierarchical clustering generates a similar pattern in which 11 ADNP cases generate one distinct cluster mainly representing hypomethylation events (epi-ADNP-1, blue-dashed rectangle), and the other 11 subjects generate a cluster different from both controls and the first cluster (epi-ADNP-2, red-dashed rectangle), showing a slightly hypermethylated pattern relative to controls. Notably, methylation changes in epi-ADNP-1 are more prominant than those in epi-ADNP-2. The top pane in the heatmap indicates the phenotype. Green, controls; purple, epi-ADNP syndrome. The heatmap color scale from blue to red represents the range of the methylation levels (beta values) between 0 and 1. **c** Evaluation of the genetic coordinates of the mutations reveals that, with the exception of one, all epi-ADNP-1 subjects have a mutation upstream c.1300, and all epi-ADNP-2 cases have mutations occurring between c.2000 and c.2340. The only exception is found for one epi-ADNP-1 patient having a mutation after c.2400. **d** A schematic representation of the mutations across the ADNP protein is presented in the bottom of the figure. Blue and red indicate the protein coordinates of the mutations related with ADNP-1 and ADNP-2 episignatures, respectively. Domains outside these two had no mutations in our cohort

Genome-wide DNA methylation analysis was performed on peripheral blood DNA from the 22 subjects with confirmed clinical and molecular diagnoses of ADNP syndrome using Illumina Infinium EPIC arrays. Following normalization and quality controls, 770,508 CpG sites (probes) were retained for analysis. A comparison was performed between these patients and 88 age- and sex-matched controls. The analysis identified 1320 probes with a minimum of 10% methylation difference between the two groups and a multiple-testing corrected *p* value < 0.01 (limma multivariable regression modeling), adjusted for blood cell type compositions. Hierarchical clustering and multiple dimensional scaling demonstrated that the selected probes separated the patients from controls. However, the ADNP cases clustered in two distinct groups with a greater distance from each other than from controls (Fig. 1). We determined that the groups did not correlate with differences in age, sex, or technical batch structure of the methylation experiment. The epigenetic clustering was shown to correlate with the positions of the mutations within *ADNP*. Samples with 5′ *ADNP* mutations (upstream of cDNA nucleotide c.1300) clustered with one sample with a 3′ deletion (c.2419_2423del). For this group, the episignature was defined as the ADNP-1 episignature and the sub-cohort defined as the epi-ADNP-1 cohort (*n* = 11). The remaining samples, with mutations occurring between c.2000 and c.2340, generated the second cluster (the ADNP-2 episignature, epi-ADNP-2 cohort, *n* = 11, Fig. 1).

**Fig. 2:**
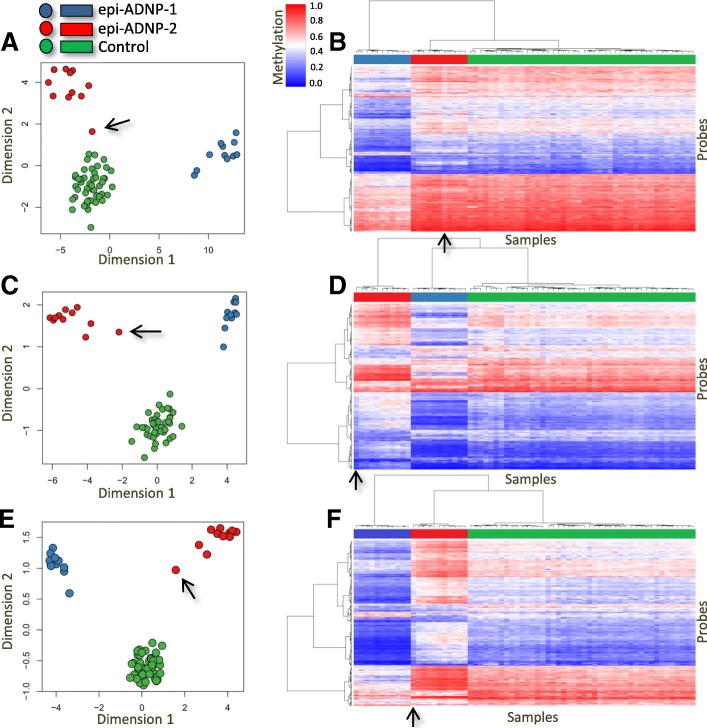
Two distinct and partially contrasting episignatures in ADNP syndrome. Separate analyses for the two identified clusters in ADNP-1 and ADNP-2 episignatures identified a larger number of probes for each group, indicating that the primary analysis had concealed the full spectrum of the methylation profiles of ADNP syndrome. Blue, red, and green (in points and panes) represent epi-ADNP-1, epi-ADNP-2, and control subjects, respectively. Using multiple dimensional scaling and hierarchical clustering analyses, it is shown that epi-ADNP-1 is associated with a mainly hypomethylated episignature (**a**, **b**). Probes associated with ADNP-1 episignature also separate epi-ADNP-2 subjects from controls, but with a milder opposite pattern of DNA methylation change (**a**, **b**). Similar observations are noted for epi-ADNP-2 specific probes (**c**, **d**), as well as the intersection of the two episignatures (**e**, **f**). The shared component (**e**, **f**) generates the most contrasting pattern between the two subtypes. Among the epi-ADNP-2 samples, a subject with a mutation in the most extreme end of the ADNP-2 region (c.2340T>G) shows the mildest changes of all (black arrows)

We split the cohort based on the two episignatures and conducted a separate analysis for each according to the same criteria described above. Comparison of the epi-ADNP-1 cohort (*n* = 11) with 44 matched controls identified 5987 differentially methylated probes—most were hypomethylated in the patients (Additional file 2: Table S1). Analysis of the 11 patients from the epi-ADNP-2 cohort matched with 44 controls identified 1374 CpG sites (Additional file 2: Table S2). Each of these two probe-sets alone was capable of distinguishing between the subjects with epi-ADNP-1, epi-ADNP-2, and controls as demonstrated using clustering analyses (Fig. 2). The ADNP-2 episignature had a smaller effect size and harbored a probe count equal to a quarter of that in the ADNP-1 episignature. Almost half the probes contributing to the ADNP-2 episignature (*n* = 541) were found to be differentially methylated in the ADNP-1 episignature. However, the methylation differences of this shared component were mostly in opposite directions. Within the ADNP-1 episignature, CpGs were mostly hypomethylated whereas they were predominantly hypermethylated in the ADNP-2 episignature (Fig. 2). In all the analyses above, a subject with a mutation in the most 3′ end of the epi-ADNP-2 cohort region (c.2340T>G; p.Tyr780*) showed the mildest ADNP-2 episignature methylation pattern, intermediate between samples from the epi-ADNP-2 cohort and controls (Fig. 2). DNA methylation analysis of whole blood specimens collected 6–9 years apart from three subjects with ADNP syndrome confirmed that the observed patterns do not change over time (Additional file 1: Figure S2).

### The two ADNP episignatures have limited overlap and differ in methylation properties

We compared the methylation profiles of the two ADNP episignatures by aligning the coordinates of the differentially methylated regions (DMRs) and assessing the direction of methylation changes. Using the DMRcate algorithm [18], we prioritized a total of 308 DMRs for the ADNP-1 episignature and 57 DMRs for the ADNP-2 episignature based on the following criteria: three or more probes less than 1 kb apart, > 10% average regional methylation change, and a false discovery rate (FDR) of < 0.01, adjusted for blood cell type compositions (Additional file 2: Tables S3–S4). The vast majority of the DMRs identified in the ADNP-1 episignature involved hypomethylation events (*n* = 293, 95%), whereas hypermethylation predominated in the ADNP-2 episignature (*n* = 30, 53%).

**Fig. 3:**
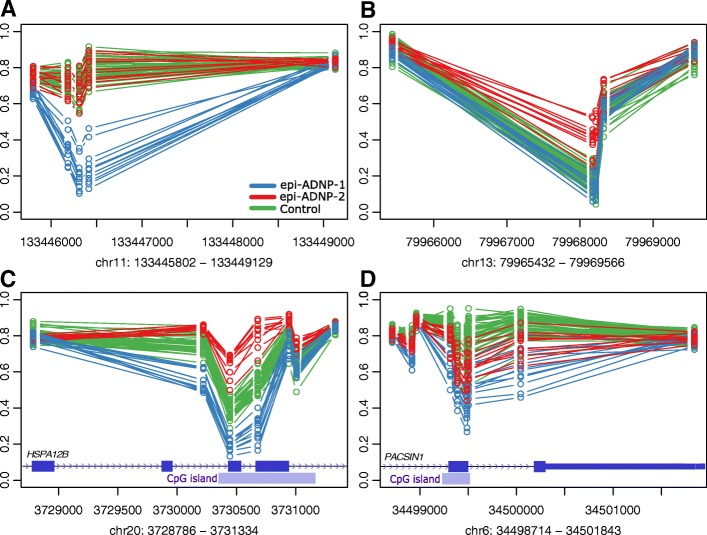
Regions differentially methylated in epi-ADNP-1 and epi-ADNP-2. Approximately 56% of the differentially methylated regions (DMRs) in epi-ADNP-1 and 26% of DMRs in epi-ADNP-2 are specific to the subtypes in which they are identified. Although the remaining DMRs are shared across the two subtypes, in the majority of instances they show contrasting methylation patterns. The small number of regions showing the same direction of change in epi-ADNP-1 and epi-ADNP-2 (< 10% of DMRs) tend to represent small levels of methylation change and do not generate fully overlapping patterns in epi-ADNP-1 and epi-ADNP-2. **a** At the most differentially methylated region (intergenic) in epi-ADNP-1 (blue), no methylation change is observed in the epi-ANDP-2 cases (red) relative to controls (green). **b** An intronic region in *RBM26* is hemimethylated in epi-ADNP-2 cases while showing a hypomethylated pattern in controls and ADNP-1. **c** A region in the gene body of *HSPA12B* represents an example of a contrasting DNA methylation change in epi-ADNP-1 and epi-ADNP-2, being hypo- and hypermethylated in each, respectively. **d** A region in the terminal end of the *PACSIN1* gene is among the very few DMRs showing a considerable methylation change in the same direction (hypomethylation) in both episignatures. In this region, however, the two subtypes are still distinguishable from each other by epi-ADNP-2 showing an intermediate pattern between epi-ADNP-1 and controls. *X*-axis, genomic coordinate; *Y*-axis, DNA methylation levels between 0 and 1; circles, DNA methylation level for every individual at one CpG site, methylation patterns in all DMRs are provided in Additional file 1: Figures S2–S14

For every region identified, we examined the methylation status in the other ADNP episignature (Additional file 2: Tables S3-S4). From the 308 DMRs in the ADNP-1 episignature, 174 (56%) were not differentially methylated in the ADNP-2 episignature. The most prominent DMRs in this category include 38% hypomethylation at chr11:133445802–133446415 (intergenic), 31% hypomethylation in chr11:13508769–13509032 (10 kb upstream *PTH*), and 29% hypomethylation in chr13:20392406–20392981 (8 kb upstream *ZMYM5*) (Fig. 3 and Additional file 1: Figure S3–S13). Among the ADNP-1 episignature DMRs, 108 (35%) showed an opposite direction, and 26 (9%) showed the same direction of methylation change in the epi-ADNP-2 cohort (Additional file 1: Figures S14–S15). Most of these changes had a smaller effect size in the epi-ADNP-2 cohort than in epi-ADNP-1 and, in many cases, did not meet the strict cut offs applied in DMR mapping. These statistics were slightly different for the ADNP-2 episignature DMRs: 15 (26%) were found to be unaffected, 33 (58%) showed a contrasting pattern, and 9 (15%) had a change in the same direction in epi-ADNP-1.

Almost all the DMR coordinates shared between the two episignatures (~ 91%) represented changes in opposite directions (i.e., hypomethylation vs. hypermethylation), and the number of changes in the same direction was low and restricted to DMRs with low effect sizes. Within this latter category (~ 9%), despite a shared direction of change, the extent of methylation difference was not similar 60% of the time, with the epi-ADNP-2 cohort most often showing an intermediate methylation level between the epi-ADNP-1 cohort and controls. The main DMRs in this category include a change in chr16:29703339–29703480 (3 probes), mapping to the gene bodies of *BOLA2* and *QPRT* (27% and 17% hypomethylation in epi-ADNP-1 and epi-ADNP-2), and another in chr6:34498714–34500043 (8 probes), encompassing a hypomethylation event in exon 9 of *PACSIN1* in epi-ADNP-1 (17%) and epi-ADNP-2 (11%, Fig. 3). These analyses indicated that the episignatures of epi-ADNP-1 and epi-ADNP-2 cohorts are two distinct and partially contrasting entities with a very small shared component.

### Genes involved in neuronal function are enriched in the ADNP-1 and ADNP-2 episignatures

To assess the functional significance of genes represented in the two methylation profiles, we performed gene-set, pathway, and protein interaction analyses on all of the genes annotating to a differentially methylated CpG identified here. Gene-set analysis identified six gene ontology (GO) terms enriched in the ADNP-1 episignature (FDR < 0.01) including cell communications, flavonoid metabolism, and synaptic signaling (Additional file 2: Table S5). No GO terms identified in the ADNP-2 episignature met the conservative FDR threshold of 0.01, likely due to the small number of genes involved. The most significant terms identified in this analysis, however, included extracellular matrix organization and central nervous system development. Additional file 2: Tables S5–S6 show all GO terms with a *p* value < 0.01 in ADNP-1 and ADNP-2 episignatures. Analysis of the combination of CpGs from the two profiles detected the same GO terms identified for ADNP-1 as the most significant biological processes (FDR < 0.01, Additional file 2: Table S7).

**Fig. 4:**
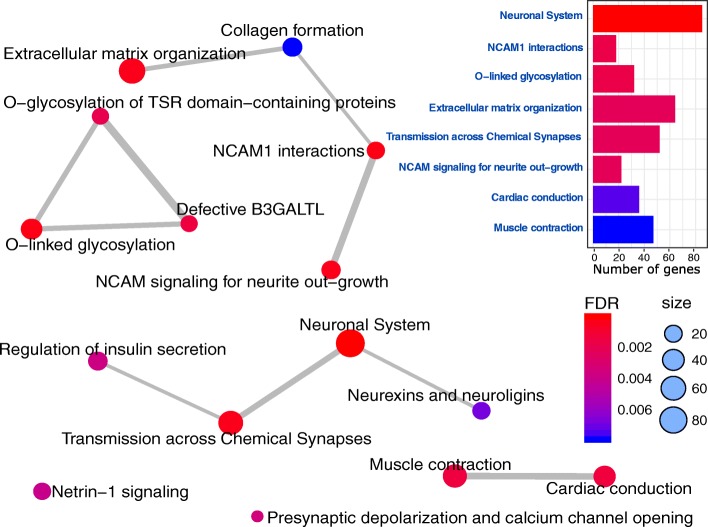
Pathways enriched in the genes in ADNP signatures. The color intensity from blue to red represents the degree of significance (*p* value). The size of each circle indicates the number of genes from each pathway that are present in the ADNP episignatures. The thickness of the connecting lines corresponds to the level of interactions and relatedness between the pathways. Neuronal system is the most significant pathway in this analysis and with the greatest number of genes (*X*-axis of the box on top right). Extracellular matrix organization and transmission across chemical synapses are the next most significant pathways. An interactive map of the genes from ADNP episignatures involved in these pathways is shown in Additional file 1: Figure S15

Pathway analysis of the ADNP-1 episignature identified 14 pathways (FDR < 0.01), the most prominent of which were neuronal system followed by extracellular matrix organization (Additional file 2: Table S8). No pathway was enriched in the ADNP-2 episignature, again, likely due to the small number of genes. Analysis of the combination of ADNP-1 and ADNP-2 episignatures retained neuronal system and extracellular matrix organization as the most significant pathways but additionally prioritized neuronal transmission across chemical synapses (Additional file 2: Table S9, Fig. 4 and Additional file 1: Figure S16). The genes involved in the neuronal system, were two-fold more likely to occur in the ADNP episignatures compared with the total number of genes tested in the EPIC array (*p* value = 7.79E− 09, FDR = 8.82E− 06).

We conducted interaction analysis of the proteins produced by genes with differentially methylated promoters using the EpiMod algorithm [34]. This analysis identified a total of nine protein–protein interaction network hotspots containing a minimum of 10 interacting partners and an FDR < 0.01 for the ADNP-1 episignature (Additional file 2: Table S10). The most active of these hotspots centered on the SFN protein. SFN had the greatest modularity index for the ADNP-1 episignature (6.98 compared to < 2.5 in all others) and was the only hotspot to meet the specified criteria in the ADNP-2 episignature. However, the predicted direction of change in gene expression for the interacting members was opposite between ADNP-1 and ADNP-2 episignatures (Additional file 2: Table S11). SFN is located at the center of an interaction network of proteins including HYAL2, NBEA, NBR1, AURKAIP1, RALGPS2, and SLC1A2, some of which have known involvement in brain function and neurological disease.

### Development of a classification model for ADNP syndrome

The presence of two distinct methylation profiles in patients with ADNP syndrome suggested that DNA methylation data could be used to develop a classification model for detection of ADNP cases and differentiation between ADNP-1 and ADNP-2 episignatures. All 22 affected patients were randomly divided into two cohorts of training (75% subset, *n* = 16) and testing (25% subset, *n* = 6). The two episignatures were equally represented in both training and testing subsets (Table 1). A sample of 64 controls was matched to the training subset for feature selection and model training. We limited the analysis to probes shared by both EPIC and 450k platforms (*n* = 399,092). Probes were filtered to those with a minimum of 10% methylation difference from controls (ADNP-1 episignature, *n* = 3876; ADNP-2 episignature, *n* = 1358). The probes differentially methylated in both ADNP-1 and ADNP-2 episignatures were expected to maximally distinguish the two groups; therefore, only shared probes were retained for feature selection (*n* = 461). From these, probes that provided the greatest separation between all three groups (ADNP-1, ADNP-2, and controls) were selected using the pairwise measurement of the area under the receiver operating characteristic curve (AUC; *n* = 163, Additional file 2: Table S12). This final probe list was used to train a multi-class support vector machine (SVM) with linear kernel on the training cohort. The model was set to generate three scores ranging from zero to one for any given subject, representing the confidence in predicting whether the subject has a DNA methylation profile resembling that in the epi-ADNP-1, epi-ADNP-2, or controls. The class obtaining the greatest score determined the episignature classification. Ten-fold cross-validation during the training process resulted in an average accuracy of 100% (model details in Additional file 2: Table S12).

**Fig. 5:**
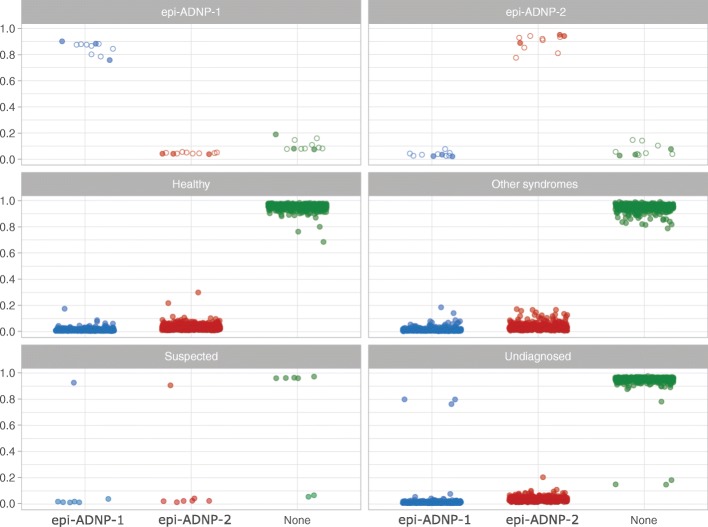
Scores generated for different subjects by the ADNP classification model. A 3-class SVM classifier generates three scores (0–1) for every subject as the probability of having a DNA methylation profile similar to what is observed in epi-ADNP-1, epi-ADNP-2, or none of these. The *Y*-axis represents scores 0–1, generated for each of the three classes on the *X*-axis. Every point represents a single sample. Hollow points indicate the training samples and filled points indicate the testing samples. By default, the SVM classifier defines a cutoff of 0.5 for assigning the class; however, the vast majority of the tested individuals received a score < 0.2 or > 0.8. Therefore, to improve visualization, the points are jittered. The first two top panels show trials performed for known cases of epi-ADNP-1 and epi-ADNP-2, all of which were classified into the correct categories. The middle two panels illustrate trials performed on 2315 healthy individuals (left) and 780 patients with neurodevelopmental syndromes other than ADNP (right), all of which are scored low for both episignatures, but have received very high scores for the non-ADNP category. This latter group includes subjects diagnosed with imprinting defects (Angelman, Prader-Willi, Beckwith-Wiedemann, and Silver-Russell syndromes), non-syndromic autism spectrum disorders, BAFopathies (Coffin-Siris, Nicolaides-Baraitser, and Chr6q25 microdeletion syndromes), RASopathies, autosomal dominant cerebellar ataxia, deafness, and narcolepsy, ATRX, Coffin-Lowry, Cornelia de Lange, CHARGE, CHOPS, Claes-Jensen, Coffin-Lowry, Down, Dup7, Floating-Harbor, Fragile X, Genitopatellar, Juberg-Marsidi, Kabuki, Rett, Saethre-Chotzen, Sotos, Weaver, and Williams syndromes. The last two panels show trials performed for suspected and unresolved cases. Among the suspected cases (*n* = 7), who based on clinical or molecular assessments are ADNP candidates, one is classified as epi-ADNP-1 and one other as epi-ADNP-2. Unresolved subjects include 1150 undiagnosed patients with neurodevelopmental presentations, among which three have been classified as epi-ADNP-1

A series of tests were performed to challenge the reliability of the model. First, the entire training cohort was classified by the model. The correct classifications were assigned to all subjects predicted to have an ADNP-1 or ADNP-2 episignature, with scores significantly different from the other two classes (Fig. 5). Next, we confirmed that the model is not sensitive to the experimental batch structure by classifying novel control samples processed on the same batch as patients. All were appropriately classified as controls. Additionally, we evaluated the extent to which the model is sensitive to variations in blood cell type composition. The model was used to classify methylation array data derived from diverse sample types from six healthy individuals, downloaded from the gene expression omnibus (GEO, GSE35069) [19]. The samples included whole blood, peripheral blood mononuclear cells, and granulocytes, as well as seven isolated cell populations (CD4+ T, CD8+ T, CD56+ NK, CD19+ B, CD14+ monocytes, neutrophils, and eosinophils) from six individuals. All of these samples were classified as controls with scores similar to those generated for the whole blood samples. The average inter-cell type variability in the scores was < 5% (Additional file 2: Table S13).

To validate the model, it was applied to the methylation data from the testing cohort, composed of three individuals known to have an ADNP-1 episignature and three with an ADNP-2 episignature. These data were completely unfamiliar to the model and had not been used for feature selection or training. All samples were assigned the expected class with scores similar to those of the training dataset, confirming that the model is robust in the classification of all three classes (Fig. 5). To measure the specificity of the classifier, we tested whole blood methylation data from 2315 healthy subjects of various racial backgrounds (aged 0–94) obtained from GEO (GSE42861, GSE85210, GSE87571, GSE87648, and GSE99863) [20–23]. All subjects were classified as controls (Fig. 5). Next, we tested whether the model could differentiate individuals with ADNP syndrome from those with other neurodevelopmental disorders. DNA methylation profiles from 780 subjects with a confirmed diagnosis of a syndromic condition, including trinucleotide repeat expansion abnormalities, imprinting defect disorders, RASopathies, BAFopathies, Mendelian disorders of the epigenetic machinery, Down syndrome, as well as 140 patients with non-syndromic autism spectrum disorders (details in Fig. 5), were supplied to the model for classification. All samples were classified as controls, further confirming the specificity of this classifier.

### Classification of subjects with an uncertain diagnosis of ADNP syndrome

We assessed the utility of this model for classifying subjects with a clinical suspicion for ADNP syndrome (Table 2, Fig. 5). First, we studied a sample from an individual with a clinical diagnosis of ADNP syndrome, but for whom genetic information was not available. This sample was classified as having the ADNP-2 episignature (ADNP-1 0.04, ADNP-2 0.90, and control 0.06), predicting that a pathogenic variant must exist between c.2000 and c.2340 in *ADNP*. Subsequent sequencing identified a nonsense variant in the expected region (c.2156dupA; p.Tyr719*; Fig. 5).

**Table 2:** Classification of uncertain cases suspected of having ADNP syndrome

ID	*ADNP* variant	In silico assessment ^d^	Population allele frequency (%)^e^	Classification (score)	Support for prediction
ADNP_18 ^a^	Not known	N/A	N/A	ADNP-2 (0.90)	^f^
ADNP_26 ^b^	c.201G>C (p.Gln67His)	Deleterious	0	ADNP-1 (0.95)	^g^
ADNP_01 ^b^	c.1039A>G (p.Met347Val)	Tolerated	0.008	None (0.96)	^h^
ADNP_06 ^b^	c.2963C>T (p.Thr988Ile)	Conflicting	0.0004	None (0.97)	^h, i^
ADNP_19 ^b^	c.356A>G (p.Lys119Arg)	Conflicting	0.007	None (0.96)	^h^
ADNP_27 ^b, c^	c.1855G>T (p.Val619Phe)	Deleterious	0.003	None (0.95)	^h, j^
ADNP_28 ^b, c^	c.1855G>T (p.Val619Phe)	Deleterious	0.003	None (0.95)	^h, j^

^a^This patient was a confirmed case of ADNP syndrome, but the mutation was not known at the time of the study. ^b^Reason for assessment was reporting of a variant of unknown clinical significance in *ADNP*. ^c^Subjects ADNP_27 and ADNP_28 are fraternal twins sharing a missense change with an unknown mode of inheritance. ^d^In Silico assessment for the suspected variant was performed using three tools: SIFT, PolyPhen, and MutationTaster. A “tolerated” or “deleterious” decision was assigned only if all three tools were in agreement with regard to the variant. ^e^Allele frequency was obtained from the gnomAD database (v2.1) and represents the combined frequencies of different subpopulations; ^f^ADNP sequencing later identified a nonsense variant in the expected ADNP-2 region: c.2156dupA (p.Tyr719*). ^g^Variant is absent from the general population and was classified as likely pathogenic according to the ACMG guidelines. ^h^Population minor allele frequency is too high for a dominant condition. ^i^Variant is inherited from an unaffected mother. ^j^No further data is available for assessment. *N/A* not applicable. ADNP transcript, NM_015339.4. Photographs of some of these subjects are provided in Additional file 1: Figure S1

**Fig. 6:**
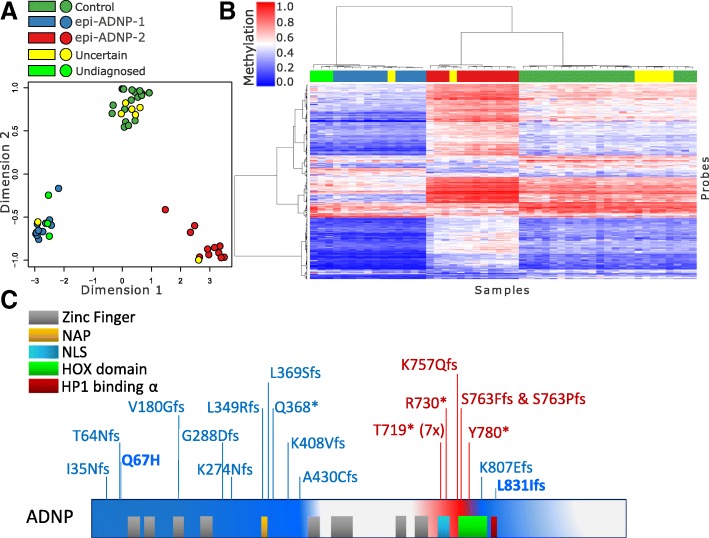
Separate evaluation of uncertain ADNP cases and those detected among unresolved patients. **a**, **b** Seven subjects with uncertain diagnoses of ADNP syndrome (yellow) together with three subjects detected in the unresolved cohort (neon green) added to the clustering analyses performed earlier in Fig. 2e and f. As expected, five of the uncertain cases are clustered with controls, one clustered with epi-ADNP-2, and one other is clustered with epi-ADNP-2 subjects (similar to the classification by our classifier). The three unresolved cases, as expected, are clustered with epi-ADNP-1 group. All of these cases show a DNA methylation pattern consistent with their respected predicted category. **c** The updated list of causative variants detected in ADNP syndrome following the assessment of unresolved/suspected subjects is illustrated in the ADNP protein

Missense variants in *ADNP* have been associated with disease in the literature [24–26]. However, this evidence may be insufficient to establish missense variation as a mechanism for disease [1, 4]. With this uncertainty, clinical labs frequently report rare missense variants in *ADNP* as uncertain significance (VUS), affecting a significant portion of patients with non-specific developmental delay (DD), ID, and/or ASD. Parental sequencing can provide further information regarding clinical significance; however, until now there have been no functional assays to aid variant interpretation. We recruited 6 subjects from our clinic and from the ADNP Kids parent support group, who had features of ADNP syndrome and a missense variant (Table 2, Fig. 5). All 6 subjects had been ascertained by clinical whole exome sequencing and received diagnostic reports listing *ADNP* variants as a potential cause of their phenotype. Two individuals were fraternal twins sharing the same missense VUS in *ADNP* (c.1855G>T; p.Val619Phe). Only one variant was confirmed to be de novo (c.201G>C; p.Gln67His). This variant affects the final nucleotide of exon 4 and is predicted to alter splicing by the online analysis tool, Human Splicing Finder [27]. The c.201G>C variant was interpreted as likely pathogenic in the exome report; all others were interpreted as VUS. All but the c.201G>C variant were present in the gnomAD database with minor allele frequencies < 0.01% [28]. Genome-wide methylation analysis classified five of the six subjects as non-ADNP. The patient with the c.201G>C variant was classified as having the ADNP-1 episignature. Separate assessments using hierarchical clustering and multiple dimensional scaling were also consistent with these findings (Fig. 6).

### Screening of unresolved DD/ID patients for ADNP syndrome

Children with DD/ID frequently go undiagnosed for a long time despite extensive diagnostic evaluation. We asked whether epigenetic analysis could identify patients affected with ADNP syndrome from a large cohort of undiagnosed patients. We screened 1150 patients in two cohorts. The first was composed of 661 subjects with developmental and intellectual disabilities with previous genetic testing but no clear molecular diagnosis. The second cohort was obtained from GEO (GSE89353) [29] and included 489 subjects with both CNV and exome sequencing assessments. These patients had various forms of syndromic and nonsyndromic DD/ID. None were suspected of having ADNP syndrome or had other confirmed genetic diagnoses. The analysis identified three subjects with ADNP-1 episignatures. Each scored > 0.75 for the ADNP-1 class (Fig. 5). Separate assessments using hierarchical clustering and multiple dimensional scaling revealed that all three cases have a DNA methylation profile consistent with the ADNP-1 episignature (Fig. 6). The first subject was initially assessed for CHARGE syndrome with clinical presentations of autism spectrum disorder together with iris and retinal colobomas, and with a non-coding VUS having been identified in the CHARGE-associated gene, *CHD7* (NM_017780.3:c.5534+16T>C). Our previous methylation analysis for CHARGE syndrome had indicated that this patient did not have a CHARGE-associated methylation profile [17]. Following the positive screen for ADNP syndrome, *ADNP* was sequenced and a pathogenic variant was detected (c.2491_2494del; p.Leu831Ilefs*82). This was the second patient with an ADNP-1 episignature whose mutation occurred after c.2340, further supporting the hypothesis that the ADNP-2 episignature is only caused by mutations between c.2000 and c.2340, and defects outside this region are associated with the ADNP-1 pattern. The second subject was obtained from GEO (GSE89353, patient ID: Proband156) for whom the only reported clinical feature was ASD, consistent with the major feature of ADNP syndrome. This subject and the third patient were not available for further assessments. These findings suggest that epigenomic profiling can be used as a screening tool for identifying ADNP syndrome cases among those with unresolved DD/ID.

## Discussion

The past 5 years have seen a rise in the use of genome-wide methylation arrays for identifying epigenetic patterns associated with rare diseases. To date, 16 syndromes have been described with epigenetic signatures [11–17, 30–32]. These profiles serve as effective adjuncts for genomic sequencing with utility in diagnosing patients, screening large cohorts, and clarifying the clinical relevance of variants of uncertain significance. In this study, we identified two distinct episignatures associated with ADNP syndrome. The classification model derived from these data predicted true positives and negatives 100% of the time, indicating specificity and sensitivity appropriate for diagnostic use. Indeed, when the model was applied to methylation data from individuals who had evaded diagnosis by traditional means, an ADNP syndrome diagnosis was predicted in three cases. Furthermore, the results support refinement of the mutational spectrum of ADNP syndrome. (1) Variants in *ADNP* that result in single amino acid substitution are unlikely to cause ADNP syndrome. (2) Frame-shift variants in all coding exons are predicted to cause ADNP syndrome. These findings have significant implications for individuals and families who have received uncertain diagnoses by genetic testing, particularly those that involve missense or synonymous changes that could affect splicing.

In addition to classifying individual samples and variants, epigenetic profiling is useful for guiding disease classification. Recently, epigenetic profiling of Coffin-Siris and Nicolaides-Baraitser syndromes, supported the grouping of these disorders into a single spectrum—the BAFopathies [15]. In the current study, we provide the first description of two discrete DNA methylation signatures arising from a single gene in a single clinical disorder. While at the present time, a clear epigenotype/phenotype correlation is not apparent, our data strongly suggest unique cellular mechanisms for the two ADNP methylation episignatures. Reprocessing previously established episignatures with larger cohorts might reveal other conditions with discrete episignatures.

The two predominantly opposite methylation signatures in ADNP syndrome (the ADNP-1 episignature is largely hypomethylated; the ADNP-2 episignature is hypermethylated) lead us to suspect that each mutation sub-group has unique cellular consequences. This genotype-epigenotype correlation is possibly a result of differences in ADNP protein fragment length or stability causing a disruption of DNA methylation in two unique ways. Truncating mutations scattered across the breadth of *ADNP* are associated with the ADNP-1 episignature. In contrast, the ADNP-2 episignature appears to be defined by a genomic motif including variants within the c.2156–2340 cDNA positions. This region is downstream of the nuclear localization signal (NLS) and overlaps the DNA-binding homeobox domain. When mutant ADNP protein is expressed in HEK293T cells, truncations in this region specifically disrupt entry into the nucleus [33]. It is therefore possible that the ADNP-2 episignature is a consequence of dominant negative protein products that enter the nucleus but are unable to bind DNA. However, the ADNP-1 episignature results are in conflict with other observations described by Cappuyns et al., who found that ADNP protein with exon-5 N-terminal mutations was degraded by the proteasome [33]. Our data demonstrate that N-terminal and C-terminal ADNP mutations affect DNA methylation in similar ways, indicating a possibility that in both cases, mutant protein reaches the nucleus.

One recent report associates ADNP with cognitive abilities related to intelligence, autism, Alzheimer’s disease, and schizophrenia [6]. Correlating data from gene expression studies with methylation patterns identified here has the potential to advance research in many areas. For instance, a hotspot linked to the protein SFN is associated with both the ADNP-1 and ADNP-2 episignatures. This correlation may help to explain the mechanism by which ADNP controls the level of the tumor suppressor protein, p53. SFN (Stratifin, also called 14–3-3σ) expression is influenced by the methylation of the 5′ coding sequence resulting in gene silencing in cancer [35]. SFN regulates the stability of p53 via degradation of MDM2 [36]. In the absence of SFN, p53 is targeted for degradation, promoting cell growth and proliferation [37]. These suggest that ADNP might influence p53 by modifying protein stability rather than activating gene expression as originally proposed [38].

Our clinical evaluation of the phenotyped cohort did not reveal that patients with the two episignatures of ADNP syndrome are separable based on clinical features (Additional file 1: Figure S1). This is undoubtedly due to the limited size of our phenotyped cohort (epi-ADNP-1 *n* = 3; epi-ADNP-2 *n* = 6). A previous study assessed genotype/phenotype correlation in a very large cohort of subjects with ADNP syndrome [4]. The authors identified some possible islands of correlation, but they did not match the coordinates defined by our two episignatures. Establishing a correlation between phenotype and epigenotype may require a larger cohort and a more uniform distribution of variants. It is also important to consider that the strongest determinants of episignature grouping—defined in DNA isolated from blood—may not have important phenotypic relevance. This does not mean that episignatures are unimportant, but rather that some “collateral” genomic marks are definitive biomarkers even if they are not causative of disease. Therefore in the case of ADNP syndrome, it may be most useful to explore the limited DMRs with shared epigenetic patterns between ADNP-1 and ADNP-2 episignatures.

One such area occurs in *PACSIN1*, which showed hypomethylation in all ADNP syndrome samples and has a considerable overlap in cellular function with ADNP. PACSIN1 is a neuron-specific member of the protein kinase C and casein kinase 2 substrate family. It interacts with dynamin and N-wasp to coordinate synaptic vesicle endocytosis and actin polymerization [39, 40], which in turn support neurogenesis and the maturation of dendritic spines [41]. PACSIN1 also interacts with tau proteins in neurons to reduce elongation and branching by facilitating microtubule instability [42]. Conversely, ADNP is required for neurite outgrowth in cell culture and the NAP peptide promotes outgrowth and branching [43]. Heterozygous ADNP knockout mice develop tauopathies, possibly due to the fact that NAP interacts with the neuronal microtubule network [44]. Given the consistently hypomethylated region of *PACSIN1* detected in the ADNP cohort and the functional overlap of these proteins, we believe that the relationship between *PACSIN1* and *ADNP* warrants further study.

## Conclusions

This study describes the first evidence of a Mendelian condition with two distinct peripheral blood episignatures caused by mutations in a single gene. These results suggest that two unique functional properties contribute to ADNP syndrome. These highly sensitive and specific DNA methylation episignatures in peripheral blood enable the diagnosis, screening, and classification of ADNP-suspected patients with genetic VUSs, and provide novel avenues for implementation of this technology in clinical diagnostic laboratories.

## Methods

### Patients and cohorts

Peripheral blood genomic DNA samples from patients with ADNP syndrome were obtained from the following sources: The Greenwood Genetic Center (Greenwood, SC, USA), collaborations established through the GeneMatcher exchange [45], and families in partnership with the ADNP Kids parent support group (https://www.adnpkids.com).

The first set of controls that were used for mapping of the episignatures, feature selection, and model training were collected from the Greenwood Genetic Center and the reference cohort in LHSC laboratory. A larger set of controls that were later used to measure the specificity of the classification model developed later in the study were compiled from five large databases of general population samples with various age and racial backgrounds (GSE42861, GSE85210, GSE87571, GSE87648, and GSE99863) [20–23].

Samples, from patients with congenital syndromes other than ADNP syndrome and those caused by mutations in other regulators of the epigenomic machinery that were only used for measuring the specificity of the classification model, comprised data described in our previous studies [11, 14–17, 46, 47] and included a large group of patients with autosomal dominant cerebellar ataxia with deafness and narcolepsy, ATRX syndrome, Claes-Jensen syndrome, CHARGE syndrome, CHOPS syndrome, Cornelia de Lange syndrome, Down syndrome, Fragile X syndrome, Floating-Harbor syndrome, Genitopatellar syndrome, Juberg-Marsidi syndrome, Kabuki syndrome, Angelman syndrome, Prader-Willi syndrome, Beckwith-Wiedemann syndrome, Coffin-Lowry syndrome, Rett syndrome, Saethre-Chotzen syndrome, Sotos syndrome, autism spectrum disorders, BAFopathies, and RASopathies. Added to this cohort were samples from patients with Silver-Russell syndrome, Weaver syndrome, Williams syndrome, and chr7q11.23 duplication syndrome, which were downloaded from gene expression omnibus (GEO–GSE104451, GSE55491, GSE74432, and GSE66552) [15, 48–50]. We supplemented the cohort of subjects with CHARGE syndrome, Sotos syndrome, Kabuki syndrome, and Down syndrome with publically available DNA methylation data from GEO (GSE74432, GSE116300, GSE97362, GSE52588) [13, 30, 51]. While all of these syndromes represent clinical features overlapping with ADNP syndrome, i.e., intellectual disability and facial dysmorphism, others are associated with specific DNA methylation patterns across the genome. We used this cohort to confirm that the DNA methylation episignature of ADNP syndrome does not overlap with other constitutional disorders.

Any subject used herein to represent a condition had a confirmed clinical diagnosis of the aforementioned syndrome and was screened for mutations in the related genes. The mutation report from every patient was reviewed according to the American College of Medical Genetics and Genomics (ACMG) guidelines for interpretation of genomic sequence variants [52], and only individuals confirmed to carry a pathogenic or likely pathogenic mutation together with the clinical diagnosis were used to represent a syndrome.

Samples with uncertain diagnoses as well as unsolved cases, which were used to assess the diagnostic potentials of the ADNP DNA methylation episignatures, were collected from all of the sources above over a period of 4 years. These samples were supplemented with publically available DNA methylation files from GEO for a cohort of unsolved subjects with neurodevelopmental disorders/congenital anomalies (GSE89353) [29].

### Methylation array and quality control

Peripheral whole blood DNA was extracted using standard techniques. Following bisulfite conversion, DNA methylation analysis of the samples was performed using the Illumina Infinium methylation 450 k or EPIC bead chip arrays (San Diego, CA), according to the manufacturer’s protocol. The resulting methylated and unmethylated signal intensity data were imported into R 3.5.1 for analysis. Normalization was performed using the Illumina normalization method with background correction using the *minfi* package [53]. Probes with detection *p* value > 0.01, those located on chromosomes X and Y, those known to contain SNPs at the CpG interrogation or single nucleotide extension, and probes known to cross-react with chromosomal locations other than their target regions were removed. Arrays with more than 5% failure probe rate were excluded from the analysis. Sex of the subjects was predicted using the median signal intensities of the probes on the X and Y chromosomes and those samples discordant between the labeled and predicted sex were not used for analysis. All of the samples were examined for genome-wide methylation density, and those deviating from a bimodal distribution were excluded. Factor analysis using a principal component analysis (PCA) was performed to examine the batch effect and identify the outliers.

### Selection of matched controls for methylation profiling

Matched controls were randomly selected for methylation profiling or feature selection. All of the ADNP samples were assayed using the EPIC array. Therefore, only controls assayed using the same platform were used for the analysis. Matching was done by age and sex using the *MatchIt* package. The control sample size was increased until both the matching quality and sample size were optimized and consistent across all analyses. This led to the determination of a control sample size four times larger than that of the cases in every analysis. Increasing the sample size beyond this value compromised the matching quality. After every matching trial, a PCA was performed to detect outliers and examine the data structures. Outlier samples and those with aberrant data structures were removed before a second matching trial was conducted. The iteration was repeated until no outlier sample was detected in the first two components of the PCA.

### DNA methylation profiling of ADNP syndrome

The analysis was performed according to our previously published protocol [15, 17, 54, 55]. The methylation level for each probe was measured as a beta value, calculated from the ratio of the methylated signals vs. the total sum of unmethylated and methylated signals, ranging between zero (no methylation) and one (full methylation). This value was used for biological interpretation and visualization. For linear regression modeling, beta values were logit transformed to M-values using the following equation: log_2_(beta/(1 − beta)). A linear regression modeling using the *limma* package [56] was used to identify the differentially methylated probes. The analysis was adjusted for blood cell type compositions, estimated using the algorithm developed by Houseman et al. [57]. The estimated blood cell proportions were added to the model matrix of the linear models as confounding variables. The generated *p* values were moderated using the *eBayes* function in the *limma* package and were corrected for multiple testing using the Benjamini and Hochberg method. Probes with a corrected *p* value < 0.01 and a methylation difference greater than 10% were considered significant. The effect size cutoff of 10% was chosen to avoid reporting of probes with low effect size and those influenced by technical or random variations as conducted in our previous studies [15, 17].

### Clustering and dimension reduction

Following every analysis, the selected probes were examined using a hierarchical clustering and a multiple dimensional scaling to examine the structure of the identified episignature. Hierarchical clustering was performed using Ward’s method on Euclidean distance by the *gplots* package. Multiple dimensional scaling was performed by scaling of the pair-wise Euclidean distances between the samples.

### Identification of the differentially methylated regions

To identify genomic regions harboring methylation changes (differentially methylated regions—DMRs), the DMRcate algorithm was used [18]. First, the *p* values were calculated for every probe using multivariable limma regression modeling. Next, these values were kernel smoothed to identify regions with a minimum of three probes no more than 1 kb apart and an average regional methylation difference > 10%. We selected regions with a Stouffer transformed false-discovery rate (FDR) < 0.01 across the identified DMRs. The analysis was performed on the same sets of cases and controls used for methylation profiling and was adjusted for blood cell type compositions.

### Gene-set and pathway enrichment analysis and identification of differential methylation interaction hotspots

Gene-set enrichment analysis was performed using the missMethyl package [58]. We identified Gene Ontology (GO) terms overrepresented in the genes associated with differentially methylated probes in ADNP syndrome, taking into account the number of CpG sites per gene. All CpG sites tested in the analysis were included as the background for the enrichment analysis. The enriched GO terms with an FDR < 0.01 were considered significant. Pathway enrichment analysis was conducted using a hypergeometric model implemented in the ReactomePA package [59]. Genes annotated to all of the probes passing quality controls in the EPIC array were used as the background. Enriched pathways with FDR < 0.01 were reported.

We used the EpiMod algorithm [34] to search for the interactome hotspots of differential promoter methylation. In this algorithm, protein expression changes are inferred according to a model of inverse association between the promoter methylation and gene expression. Among the differentially expressed genes in an interactive network, a hotspot (epigenetic module (EpiMods)) is a sub-network with an exceptionally large average edge-weight density (combined methylation statistics of the neighboring genes) as compared to the rest of the network [34]. To assign a statistical significance to the identified hotspots, 1000 Monte Carlo randomization of the molecular profiles was conducted as suggested by the algorithm. Interactive network hotspots composed of at least ten genes and FDR < 0.01 were reported.

### Construction of a classification model for ADNP syndrome

To construct a classification model for ADNP syndrome, subjects were divided into training (75%) and testing (25%) cohorts, ensuring that the two ADNP subtypes later identified were equally represented in both of the training and testing cohorts. For each of the subtypes in the training cohort, a matched group of controls with a sample size of four times larger was selected. Given the majority of the samples to be tested later were assayed using 450k array, we limited the analysis to probes shared by both array types. In order to avoid the use of probes with low effect size and those susceptible to technical variation, we further restricted the probes showing a minimum of 10% methylation difference between each ADNP subtype and controls. The intersection of the two lists was used for feature selection. Using the *filterVarImp* function in the *Caret* package [60], for each probe we measured pairwise area under the receiver operating characteristic curve (AUC) between each of the two subtypes and controls. Probes that obtained the maximum AUC in all three pairwise iterations were selected (all three variable importance measures = 1). This final probe list was used to train a multi-class support vector machine (SVM) with linear kernel on the training cohort. Training was done using the *e1071* R package. To determine the best hyperparameter used in linear SVM (cost—C), and to measure the accuracy of the model, 10-fold cross-validation was performed during the training. In this process, the training set was randomly divided into ten folds. Nine-fold was used for training the model and one fold for testing. After 10-fold repeating of this iteration, the mean accuracy was calculated, and the hyperparameters with the most optimal performance were selected. For every subject, the model was set to generate three scores ranging 0–1, representing the confidence in predicting whether the subject has a DNA methylation profile similar to ADNP-1, ADNP-2, or controls. Conversion of SVM decision values to these scores was done according to the Platt’s scaling method [61]. The class obtaining the greatest score determined the predicted phenotype. The final model was applied to both training and testing datasets to ensure the success of the training.

### Validation of the classification model

We ensured that the model is not sensitive to the batch structure of the methylation experiment by applying it to all of the samples assayed on the same batch as the patients used for training. To confirm that the classifier is not sensitive to the blood cell type compositions, we downloaded methylation data from isolated cell populations of healthy individuals from GEO (GSE35069) [19], supplied them to the classification model for prediction, and examined the degree to which the scores were varied across different blood cell types. Next, the model was applied to the testing cohort (25% subset of the patients not used for feature selection or training) to evaluate the predictive ability of the model on affected subjects. To determine the specificity of the model, we supplied a large number of DNA methylation arrays from healthy subjects to the model. To understand whether this model was sensitive to other medical conditions presenting with developmental delay and intellectual disabilities, we tested a large number of subjects with a confirmed clinical and molecular diagnosis of such syndromes by the model.

### Screening of undiagnosed and uncertain cases

The finally confirmed model was used to classify subjects suspected of having ADNP syndrome including those with no sequence variant or with variants of unknown significance (VUS). In addition, we used the model to screen a large group of individuals with various forms of neurodevelopmental presentations but no established diagnosis despite routine clinical and molecular assessments. The subjects that were predicted to have any of the ADNP subtypes were evaluated based on both the clinical and molecular information. Wherever a sequence variant was found, in silico assessment was performed to provide support for the predictions using *SIFT*, *PolyPhen*, and *MutationTaster* [62–64].

## Additional files

Additional file 1: Figure S1. Facial features of individuals with the ADNP syndrome. Figure S2. Methylation patterns of specimens collected years apart in three subjects. Figure S3. DMRs differentially methylated in ADNP-1 (1–30). Figure S4. DMRs differentially methylated in ADNP-1 (31–60). Figure S5. DMRs differentially methylated in ADNP-1 (61–90). Figure S6. DMRs differentially methylated in ADNP-1 (91–120). Figure S7. DMRs differentially methylated in ADNP-1 (121–150). Figure S8. DMRs differentially methylated in ADNP-1 (151–180). Figure S9. DMRs differentially methylated in ADNP-1 (181–210). Figure S10. DMRs differentially methylated in ADNP-1 (211–240). Figure S11. DMRs differentially methylated in ADNP-1 (241–270). Figure S12. DMRs differentially methylated in ADNP-1 (271–300). Figure S13. DMRs differentially methylated in ADNP-1 (301–308). Figure S14. DMRs differentially methylated in ADNP-2 (1–30). Figure S15. DMRs differentially methylated in ADNP-2 (31–57). Figure S16. Interactive networks of genes from the ADNP episignatures. (DOCX 4248 kb)

Additional file 2: Table S1. CpG sites differentially methylated between ADNP-1 and controls. Table S2. CpG sites differentially methylated between ADNP-2 and controls. Table S3. Differentially methylated regions in ADNP-1. Table S4. Differentially methylated regions in ADNP-2. Table S5. Gene ontology (GO) terms in ADNP-1 (green: FDR < 0.01). Table S6. Gene ontology (GO) terms in ADNP-2. Table S7. Gene ontology (GO) terms in ADNP-1 and ADNP-2. Table S8. Pathways enriched in ADNP-1 episignature. Table S9. Pathways enriched in ADNP-1 and ADNP-2 episignatures. Table S10. EpiMods of DNA methylation protein-protein interactions in ADNP-1. Table S11. EpiMods of DNA methylation protein-protein interactions in ADNP-2. Table S12. Details of the SVM classifier. Table S13 Scores generated for various blood cells. (XLSX 910 kb)

## Abbreviations

ACMG: American College of Medical Genetics and Genomics
ADNP: Activity-dependent neuroprotective protein
ASD: Autism spectrum disorder
AUC: Area under the curve
BAF: BRG1/Brm-associated factor
DD: Developmental delay
DMR: Differentially methylated region
FDR: False discovery rate
GEO: Gene expression omnibus
GO: Gene ontology
ID: Intellectual disability
NLS: Nuclear localization signal
PCA: Principal component analysis
SVM: Support vector machine
VUS: Variant(s) of uncertain significance
