# Supplementary material for: Gene domain-specific DNA methylation episignatures highlight distinct molecular entities of ADNP syndrome
Source: Clin Epigenetics. 2019 Apr 27;11:64. doi: 10.1186/s13148-019-0658-5 (PMC6487024; doi:10.1186/s13148-019-0658-5)
Supplement: Supplementary file 1 — Figure S1. Facial features of individuals with the ADNP syndrome. Figure S2. Methylation patterns of specimens collected years apart in three subjects. Figure S3. DMRs differentially methylated in ADNP-1 (1–30). Figure S4. DMRs differentially methylated in ADNP-1 (31–60). Figure S5. DMRs differentially methylated in ADNP-1 (61–90). Figure S6. DMRs differentially methylated in ADNP-1 (91–120). Figure S7. DMRs differentially methylated in ADNP-1 (121–150). Figure S8. DMRs differentially methylated in ADNP-1 (151–180). Figure S9. DMRs differentially methylated in ADNP-1 (181–210). Figure S10. DMRs differentially methylated in ADNP-1 (211–240). Figure S11. DMRs differentially methylated in ADNP-1 (241–270). Figure S12. DMRs differentially methylated in ADNP-1 (271–300). Figure S13. DMRs differentially methylated in ADNP-1 (301–308). Figure S14. DMRs differentially methylated in ADNP-2 (1–30). Figure S15. DMRs differentially methylated in ADNP-2 (31–57). Figure S16. Interactive networks of genes from the ADNP episignatures. (DOCX 4248 kb) [file 13148_2019_658_MOESM1_ESM.docx]

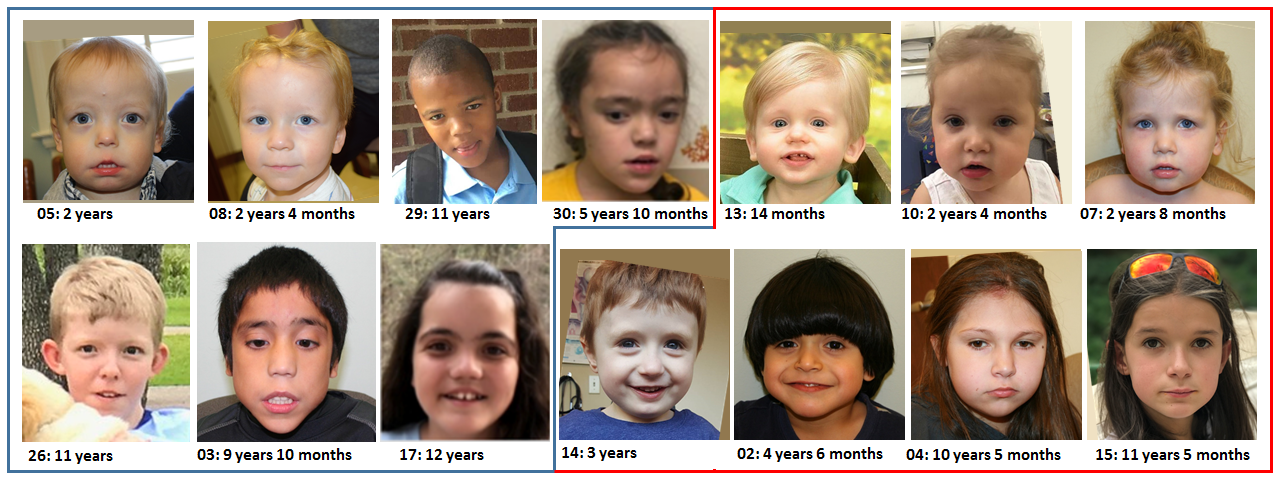


Figure S1- Facial features of individuals with the ADNP syndrome

Photographs of individuals with ADNP syndrome are displayed when available. The two digit numbers under every face indicate the patient ID’s from Tables 1 and 2 in the manuscript. Year and month indicate the age at which the picture was taken. Our study includes a patient (patient ADNP_03) with a novel mutation in *ADNP* (c.103dupA (p.Ile35Asnfs*5)). He presented with ID, severe DD, anxiety, and ASD. He was nonverbal, had self-biting violent behavior, distinctive rapid breathing, flapping of the hands and feet, and nervous laughter. Blue and red lines separate the epi-ADNP-1 and epi-ADNP-2 categories from each others, respectively (idenftified through methylation analysis – details in the results).


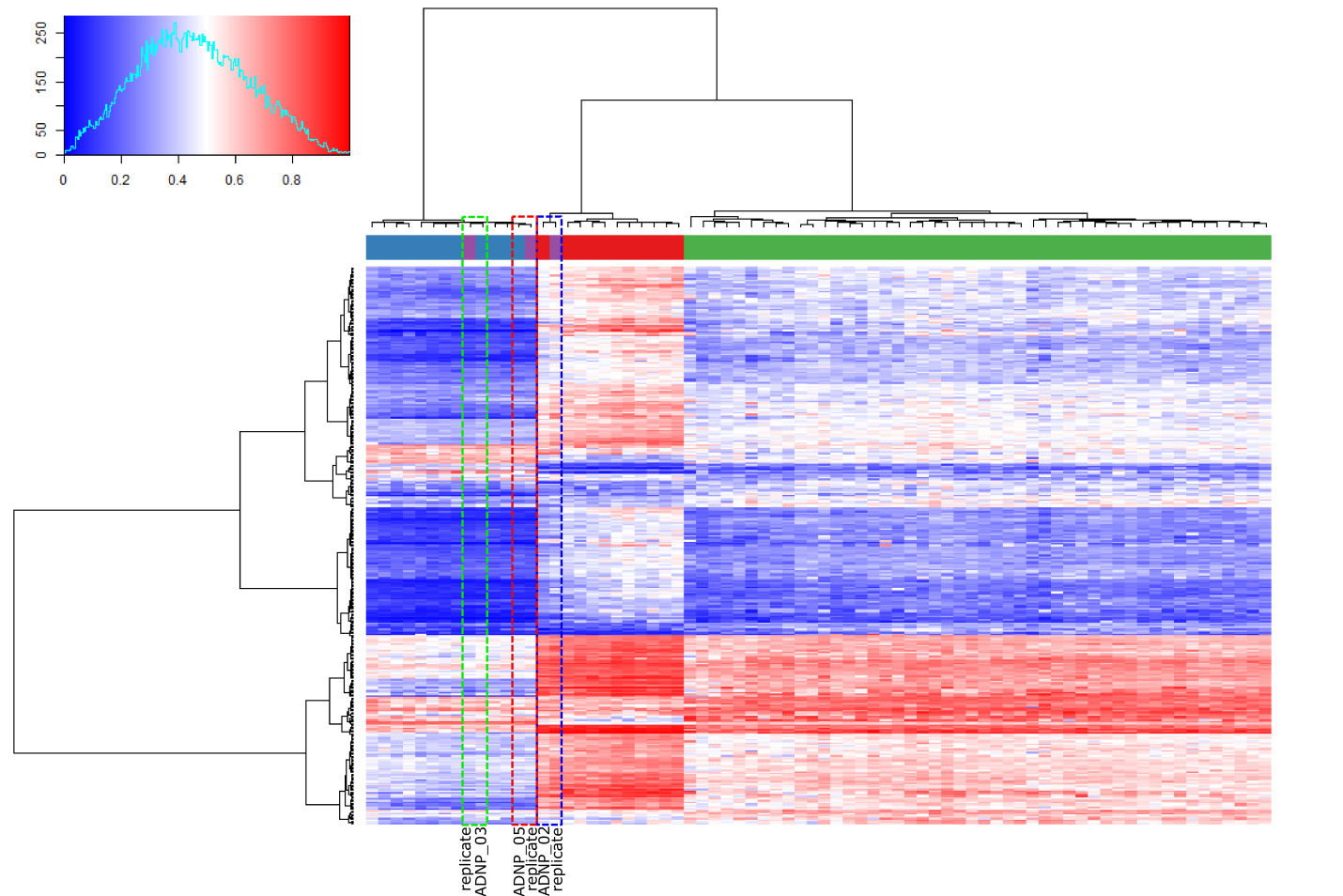


Figure S2- Methylation patterns of specimens collected years apart in three subjects

Replicate analysis of samples collected several years apart from three subjects indicate that each replicate produces a pattern very similar to the original experiment and is clustered immediately together with the other sample from the same patient. Of note, replicate of sample ADNP_02, despite being assayed using a different platofrm (450k, vs. EPIC in others), is still clustered jointly with ADNP_02. This sample has a mutation at the most extreme end of the ADNP-2 region and shows the mildest ADNP-2 DNA methylation pattern, which is also observed in its replicate.


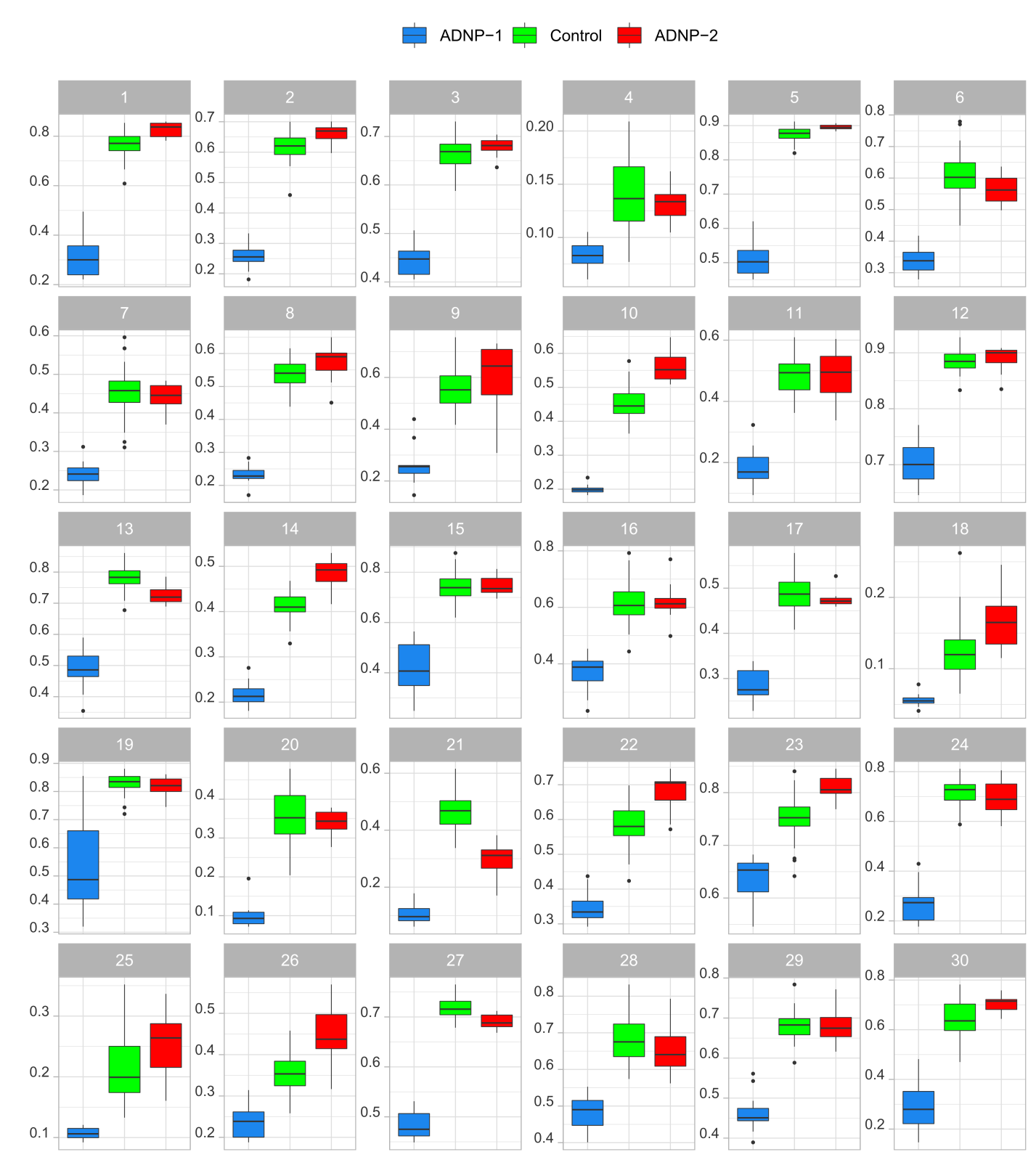


Figure S3- DMRs differentially methylated in ADNP-1 (1 – 30)

The box plots represent the distribution of median methylation values across all of the probes mapping to each region (identified in the comparison of ADNP-1 and controls) stratified by the class, i.e., Controls, ADNP-1, and ADNP-2. Centre line: median of regional methylation levels across samples; Lower and upper bounds: first and third quartiles; Whiskers: Interquartile ranges; Numbers indicate the region number from Table S3.


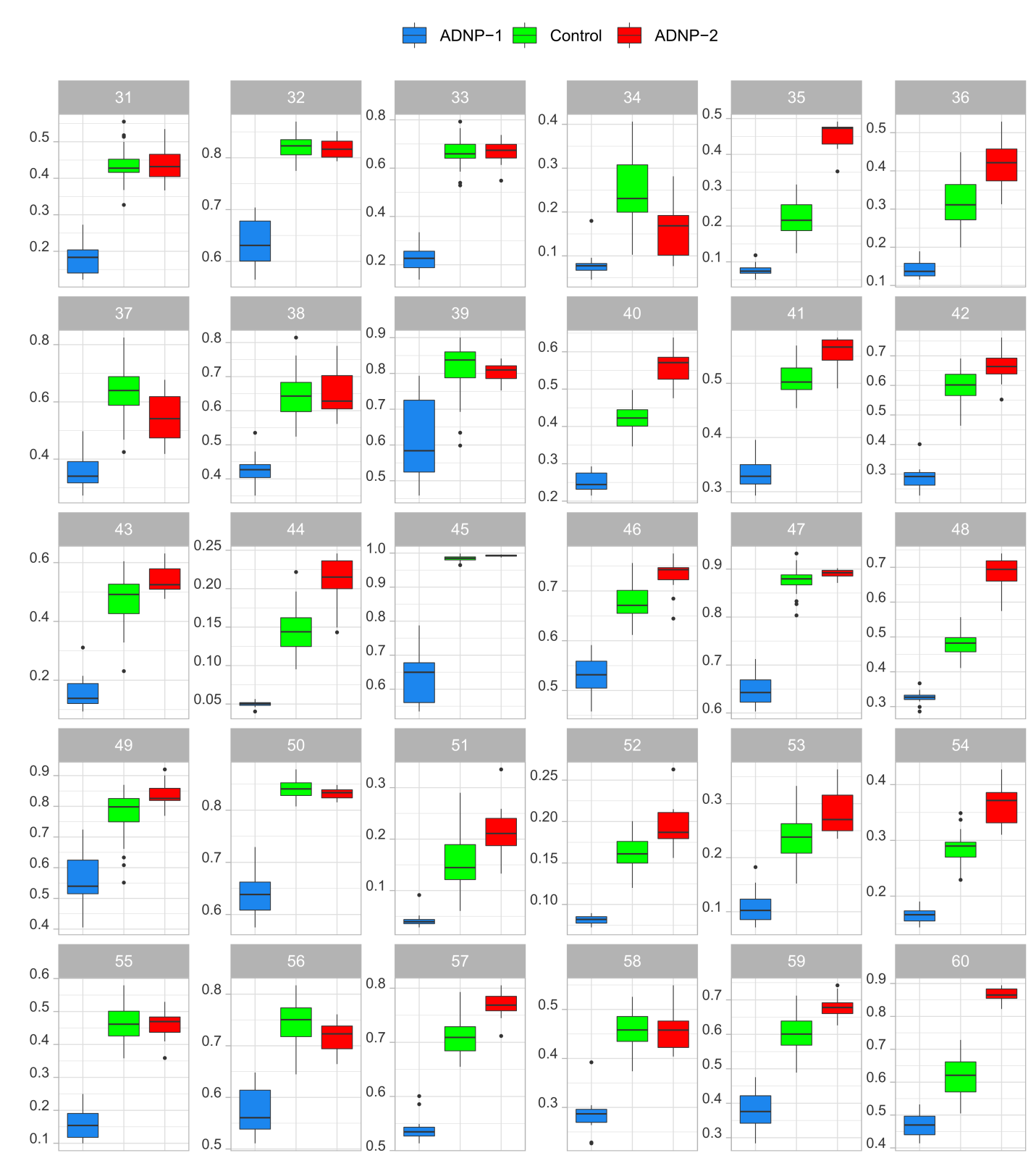


Figure S4- DMRs differentially methylated in ADNP-1 (31 – 60)


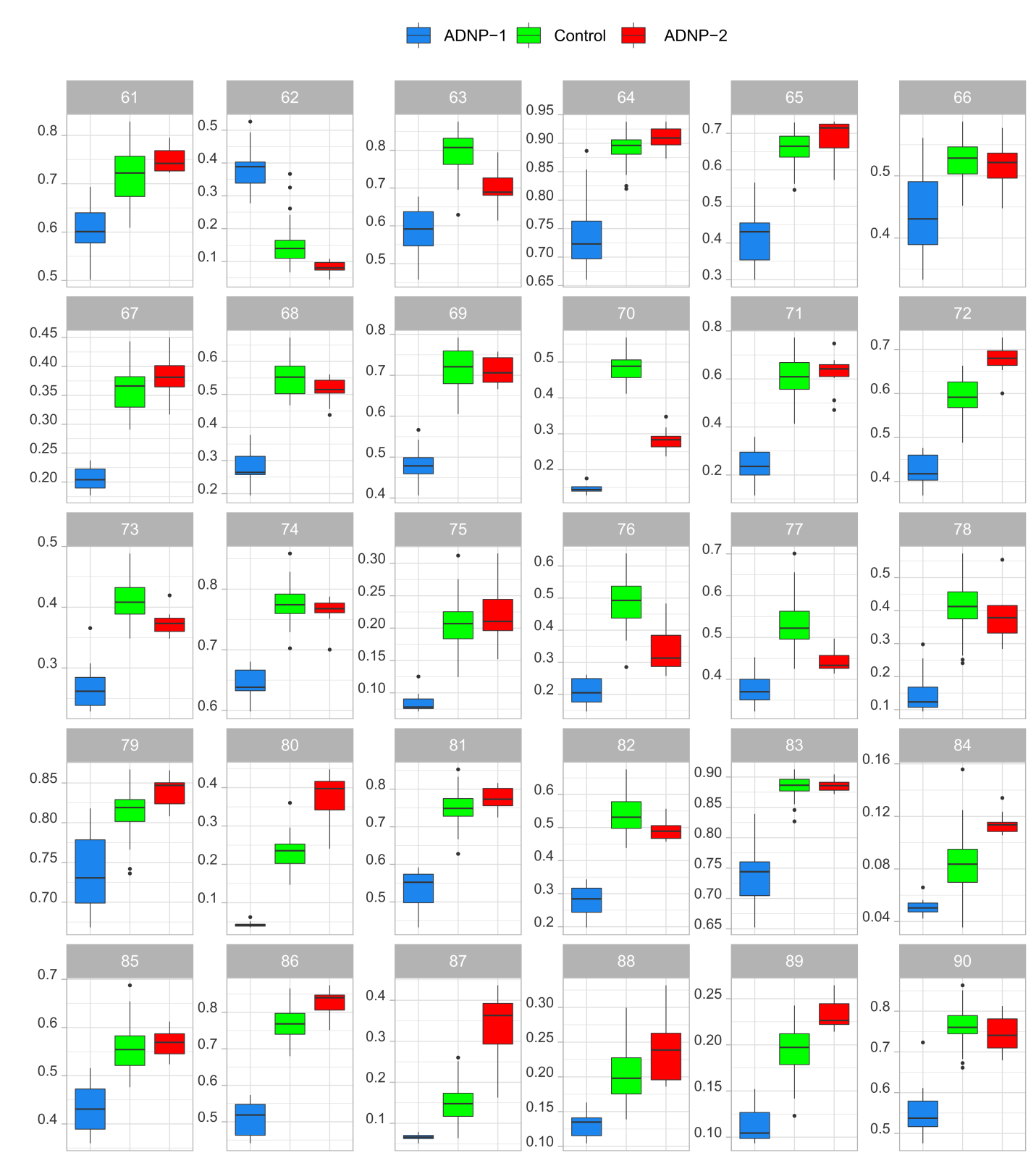


Figure S5- DMRs differentially methylated in ADNP-1 (61 – 90)


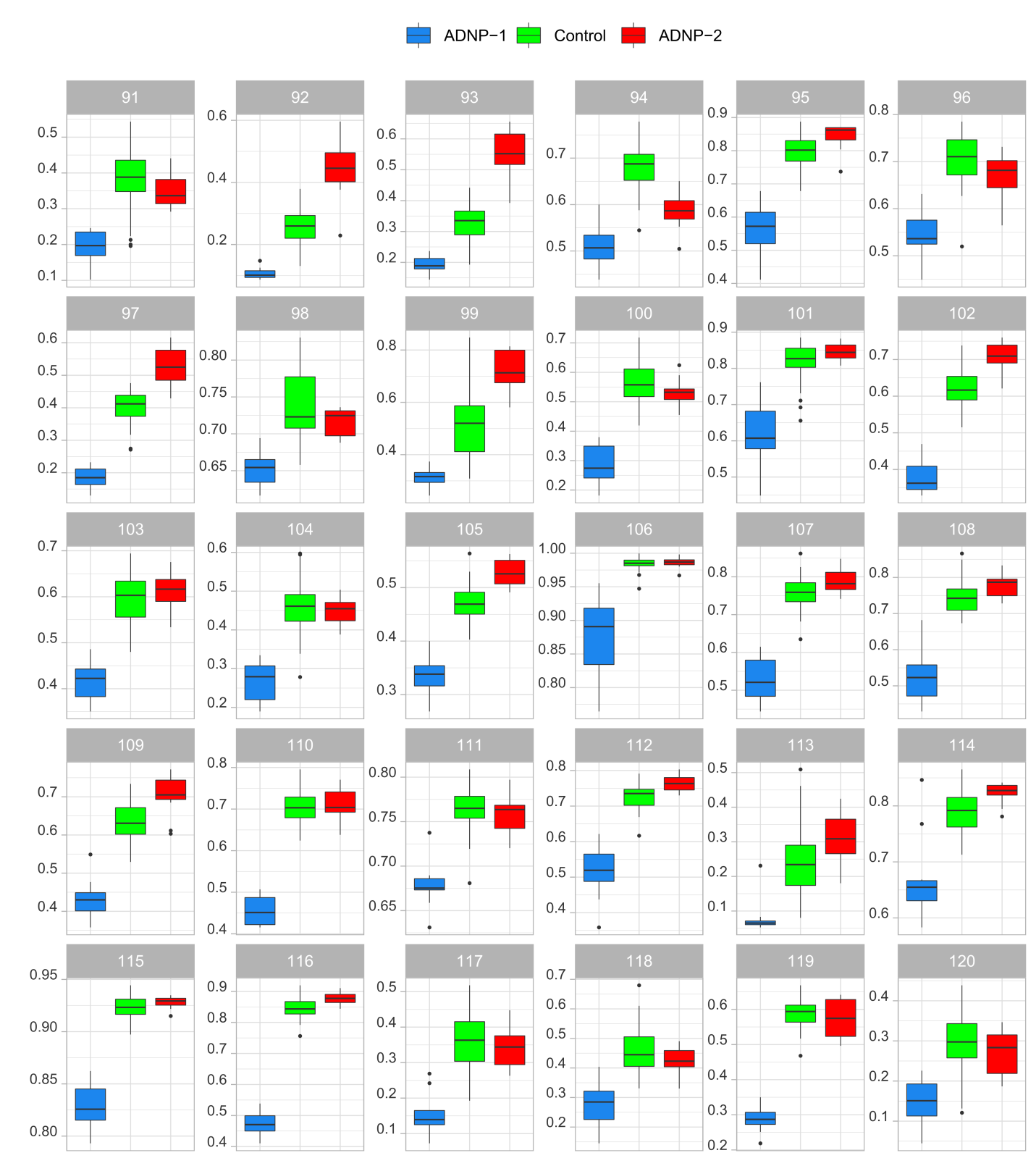


Figure S6- DMRs differentially methylated in ADNP-1 (91 – 120)


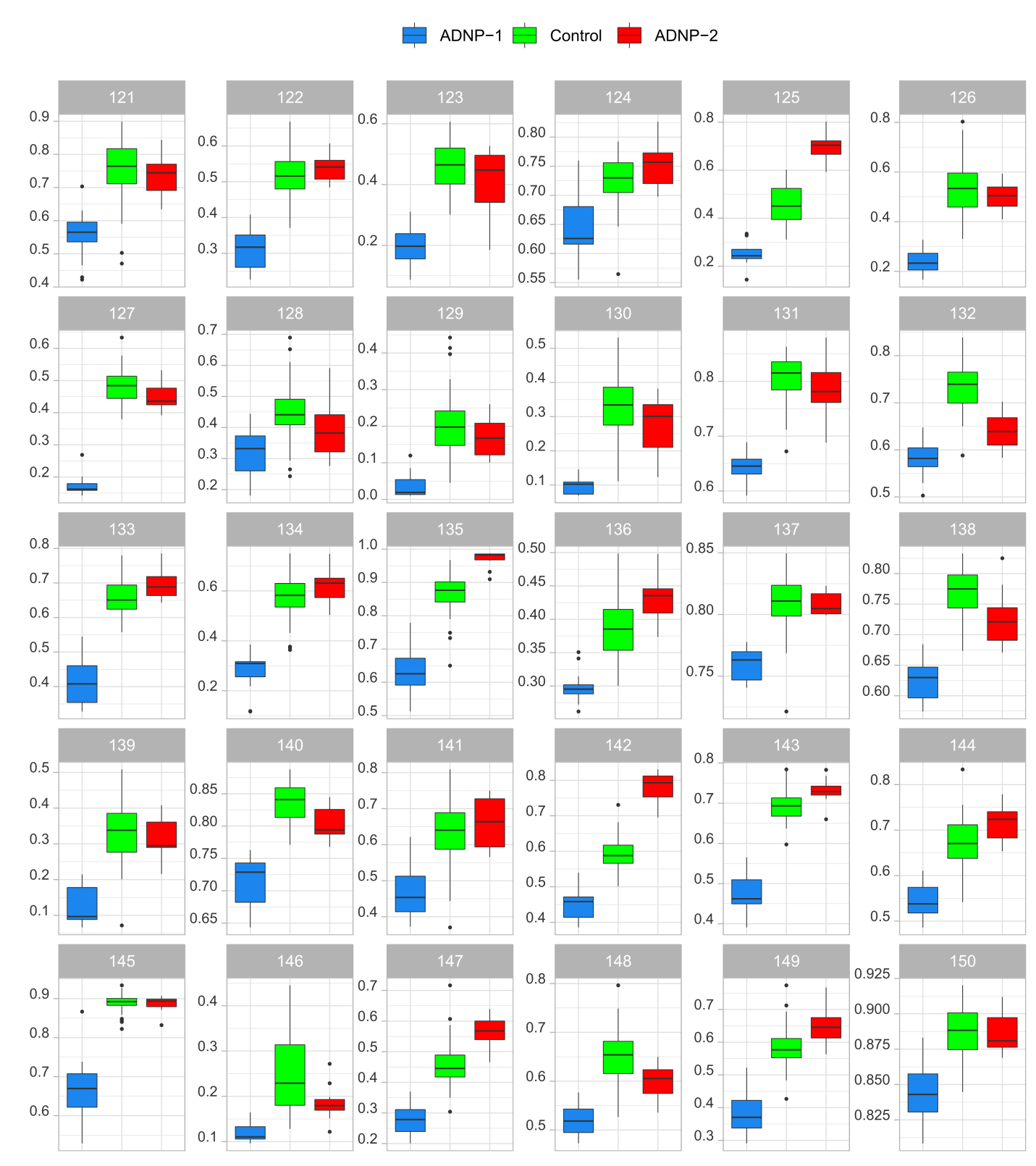


Figure S7- DMRs differentially methylated in ADNP-1 (121 – 150)


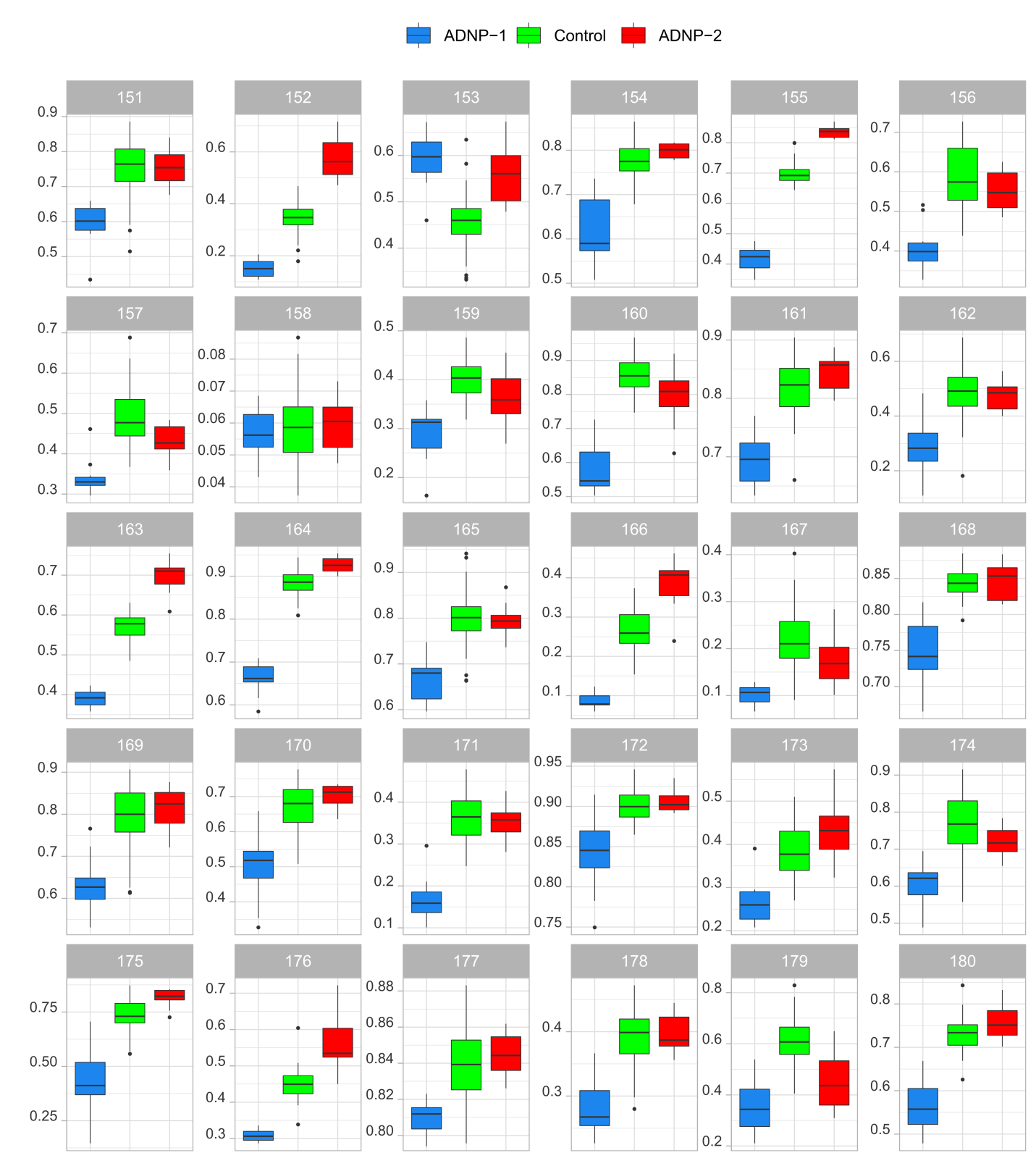


Figure S8- DMRs differentially methylated in ADNP-1 (151 – 180)


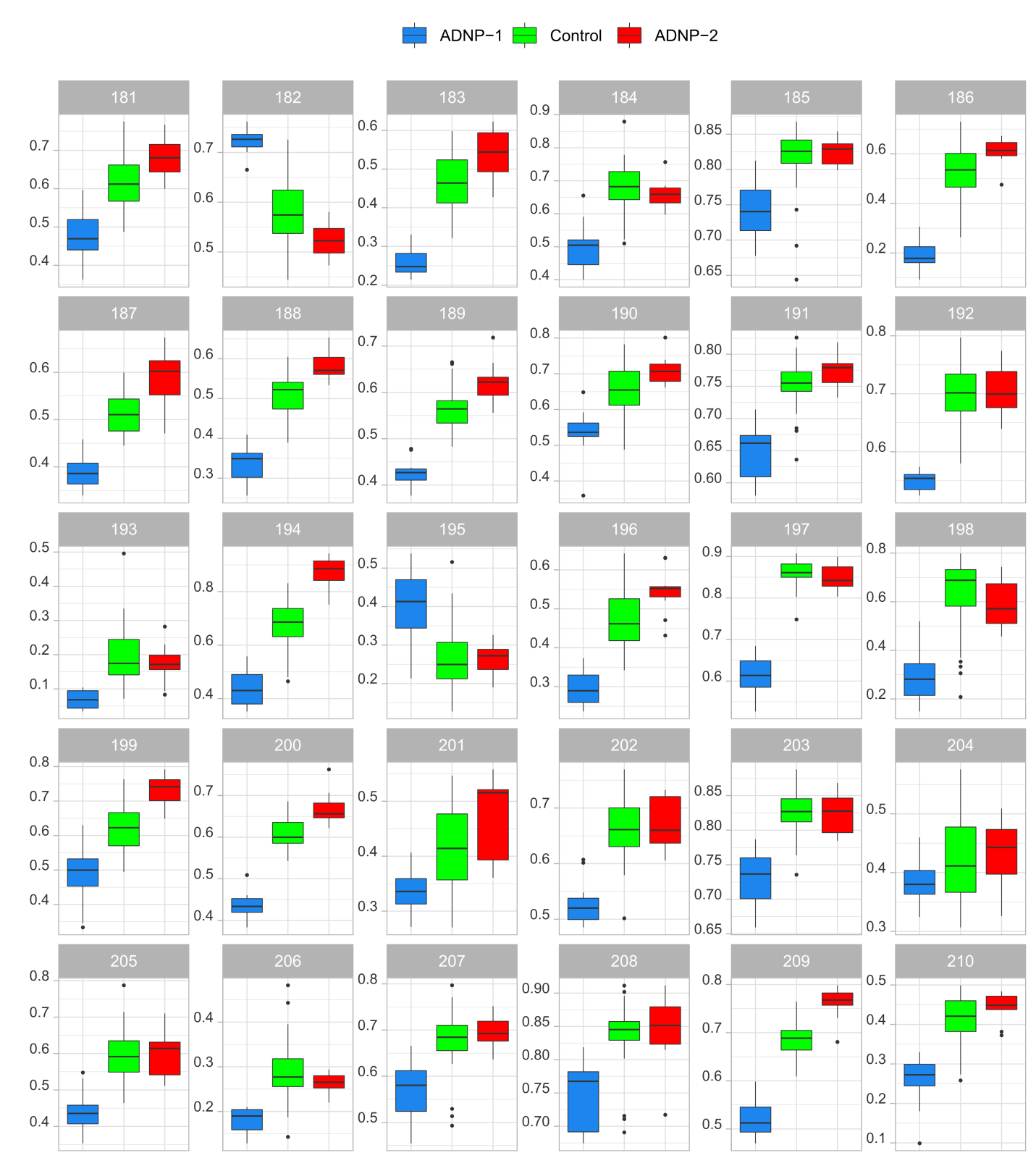


Figure S9- DMRs differentially methylated in ADNP-1 (181 – 210)


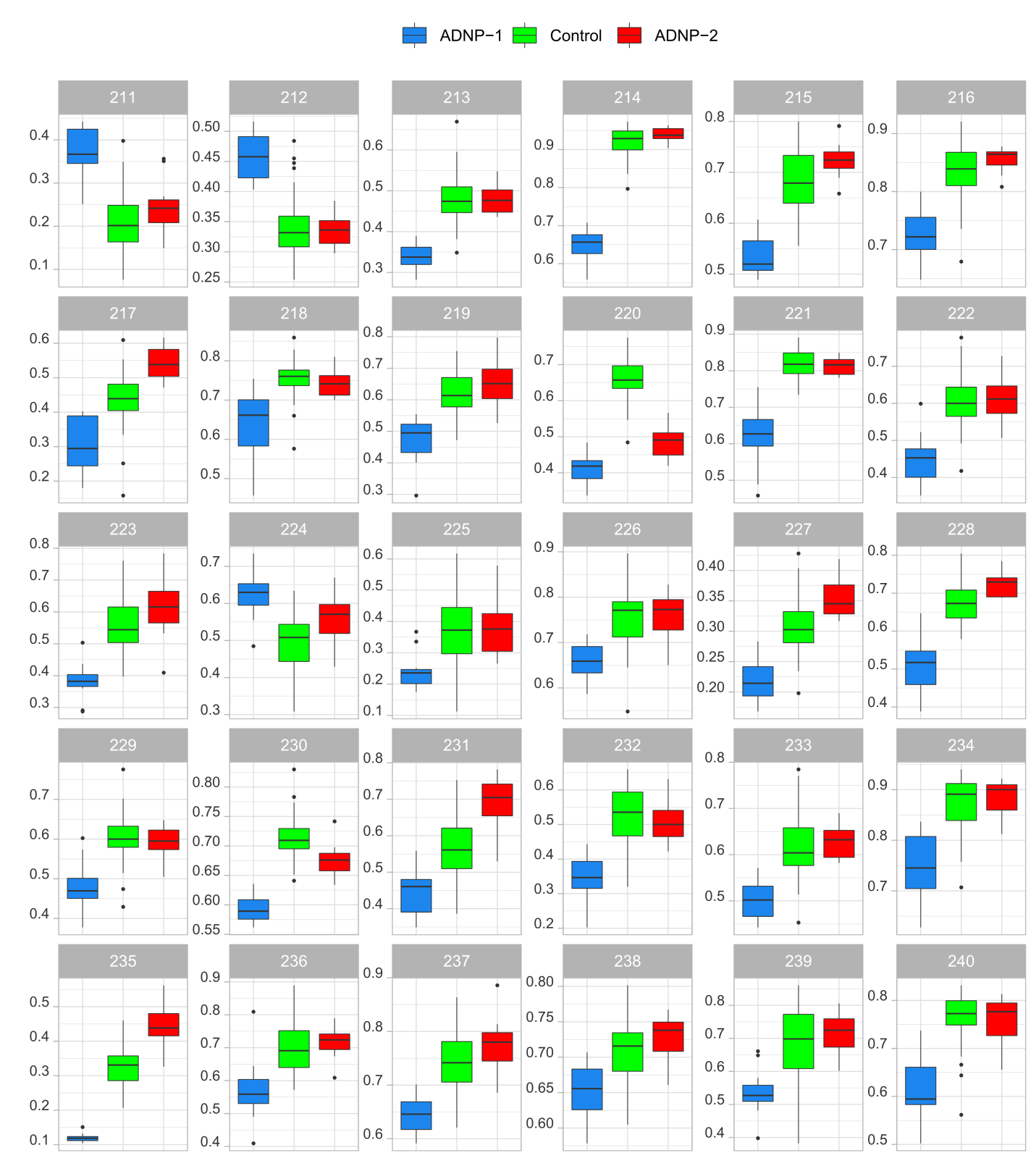


Figure S10- DMRs differentially methylated in ADNP-1 (211 – 240)


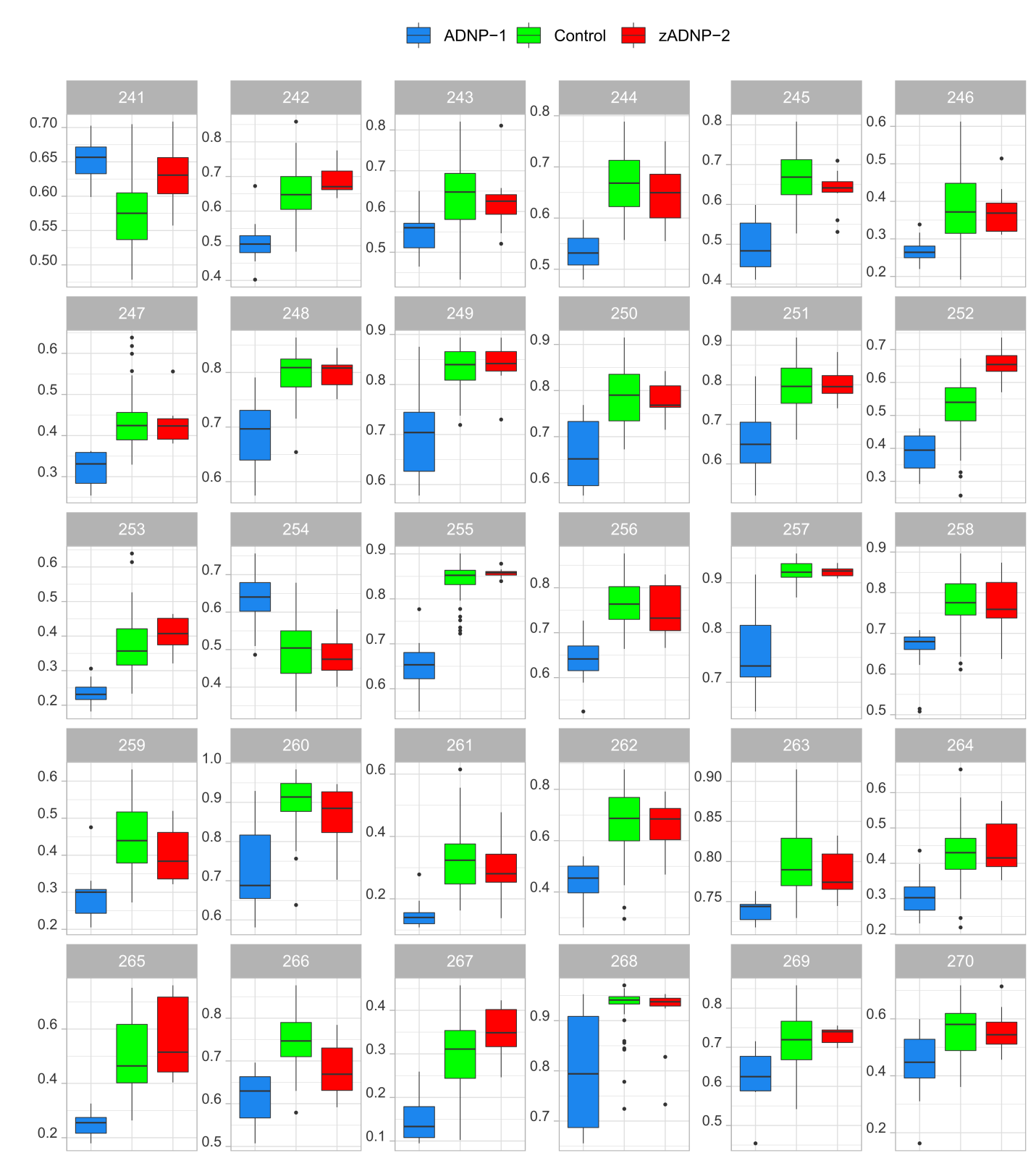


Figure S11- DMRs differentially methylated in ADNP-1 (241 – 270)


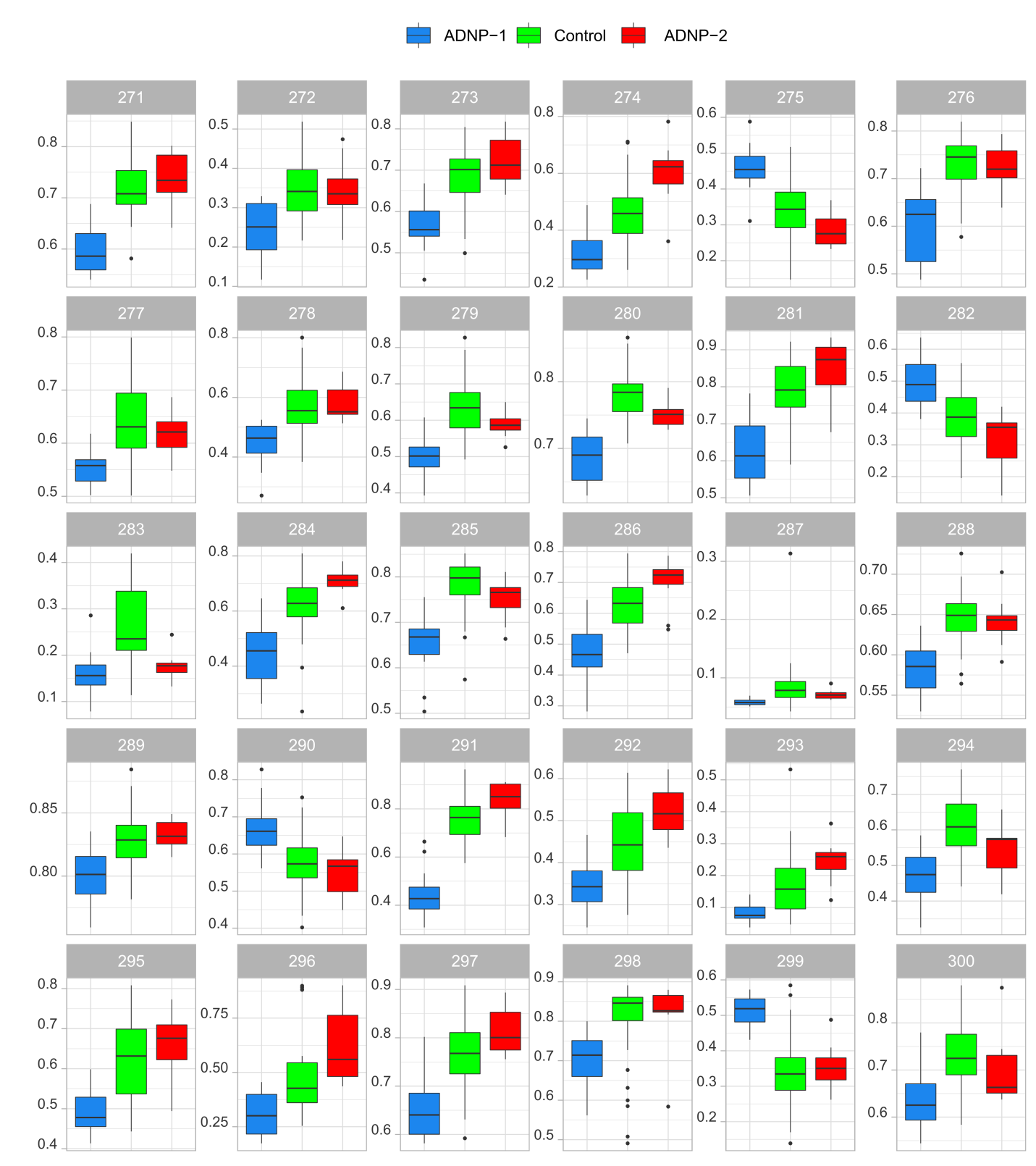


Figure S12- DMRs differentially methylated in ADNP-1 (271 – 300)


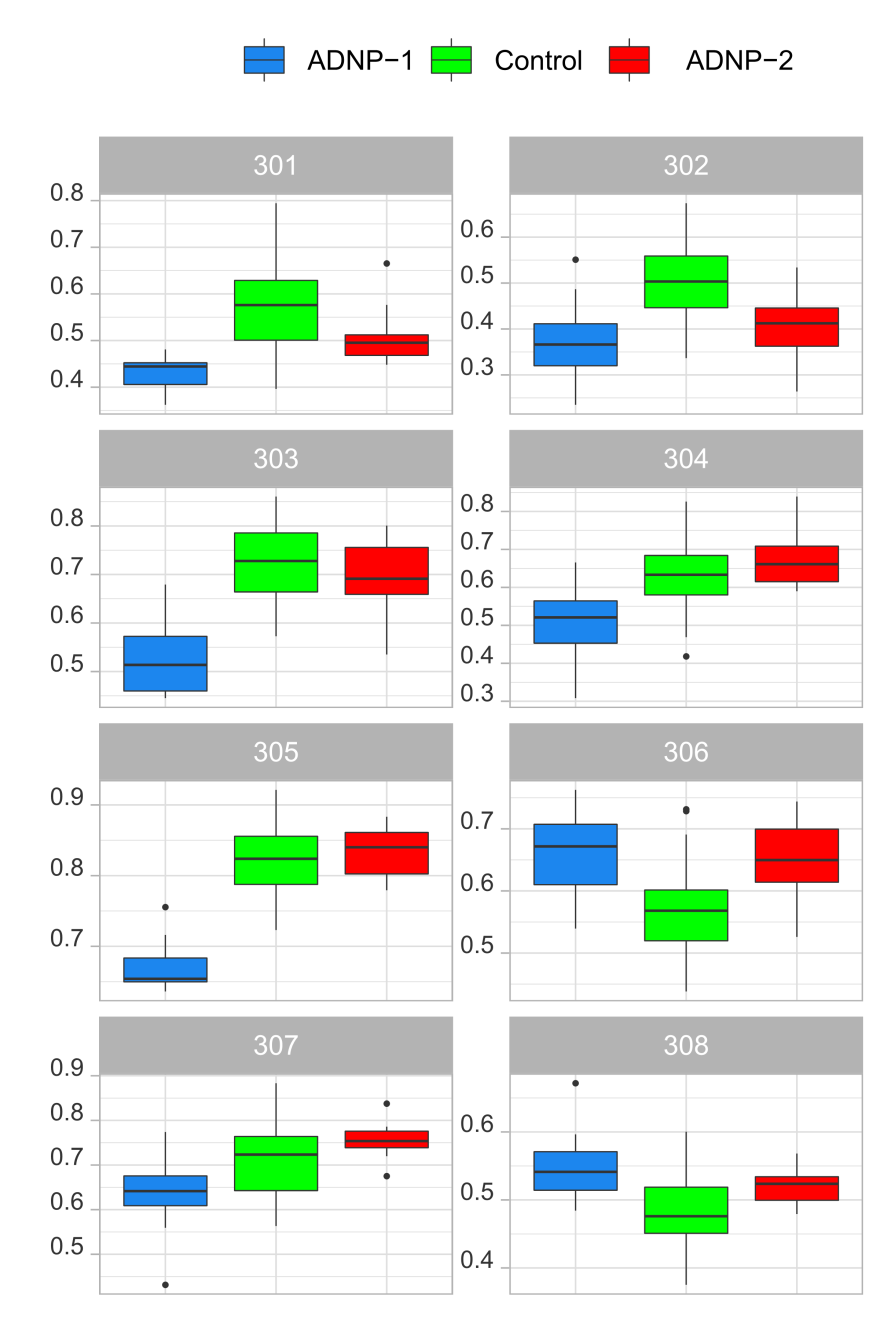


Figure S13- DMRs differentially methylated in ADNP-1 (301 – 308)


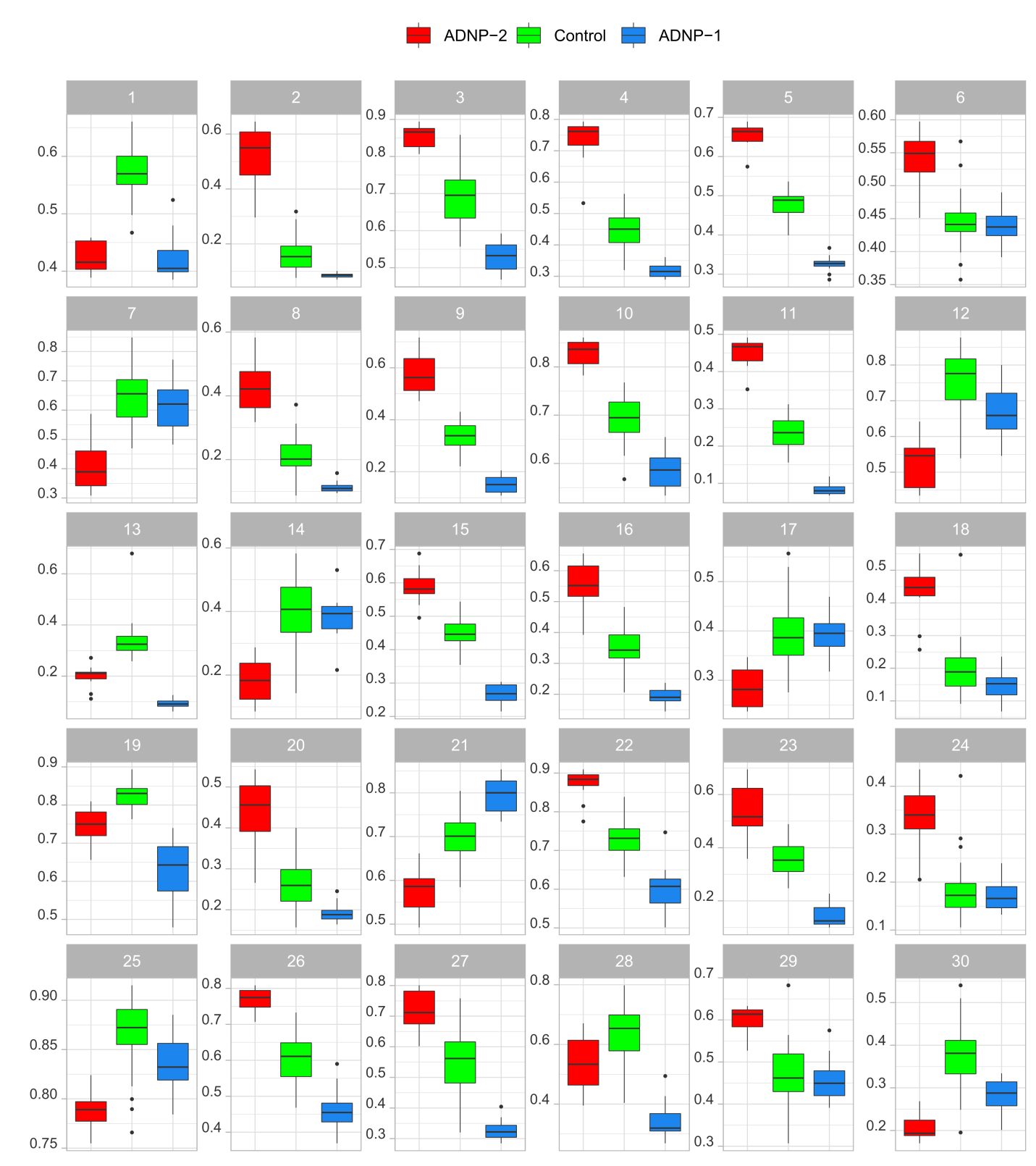


Figure S14- DMRs differentially methylated in ADNP-2 (1 - 30)

Numbers indicate the region number from Table S4.


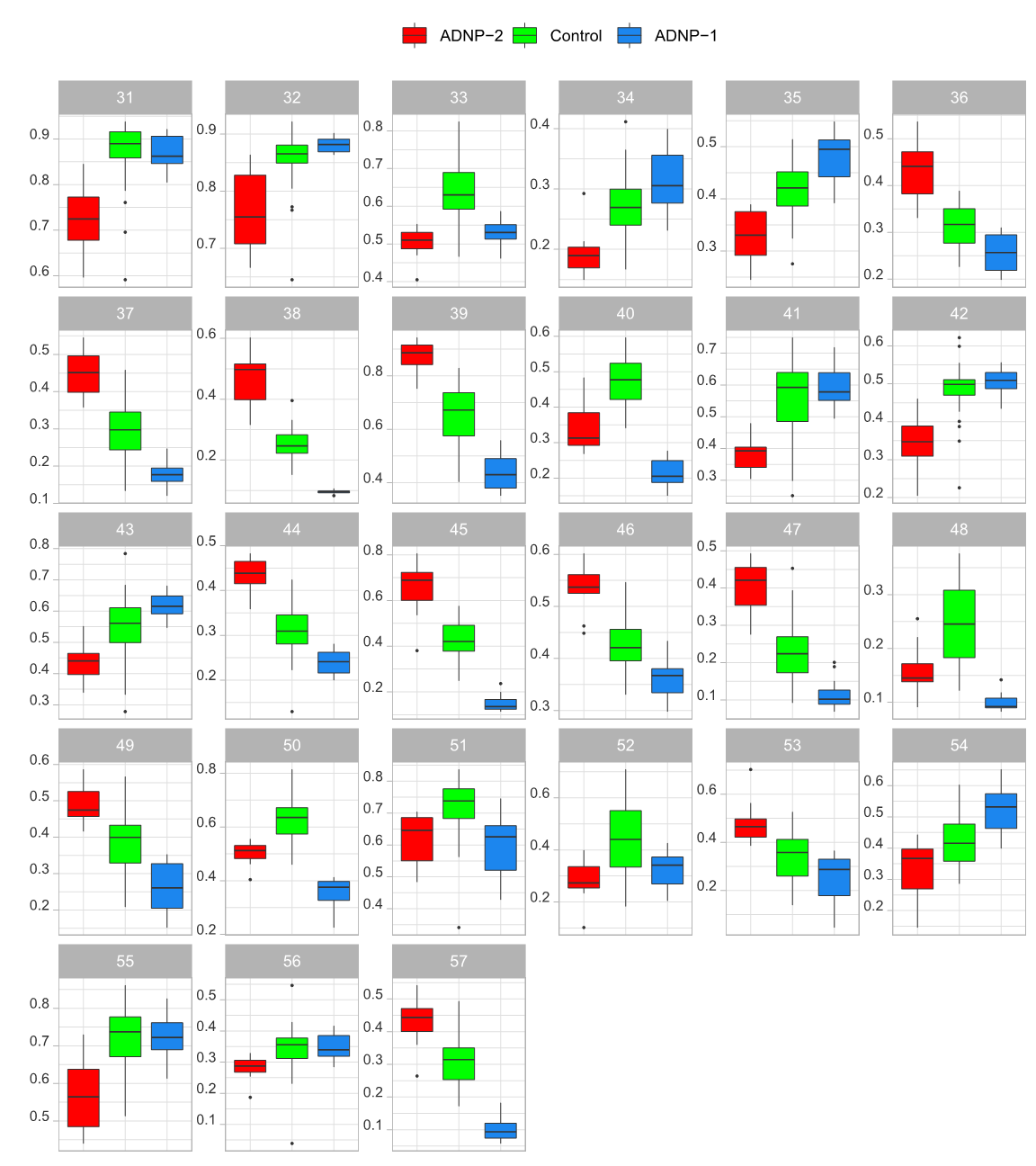


Figure S15- DMRs differentially methylated in ADNP-2 (31 – 57)


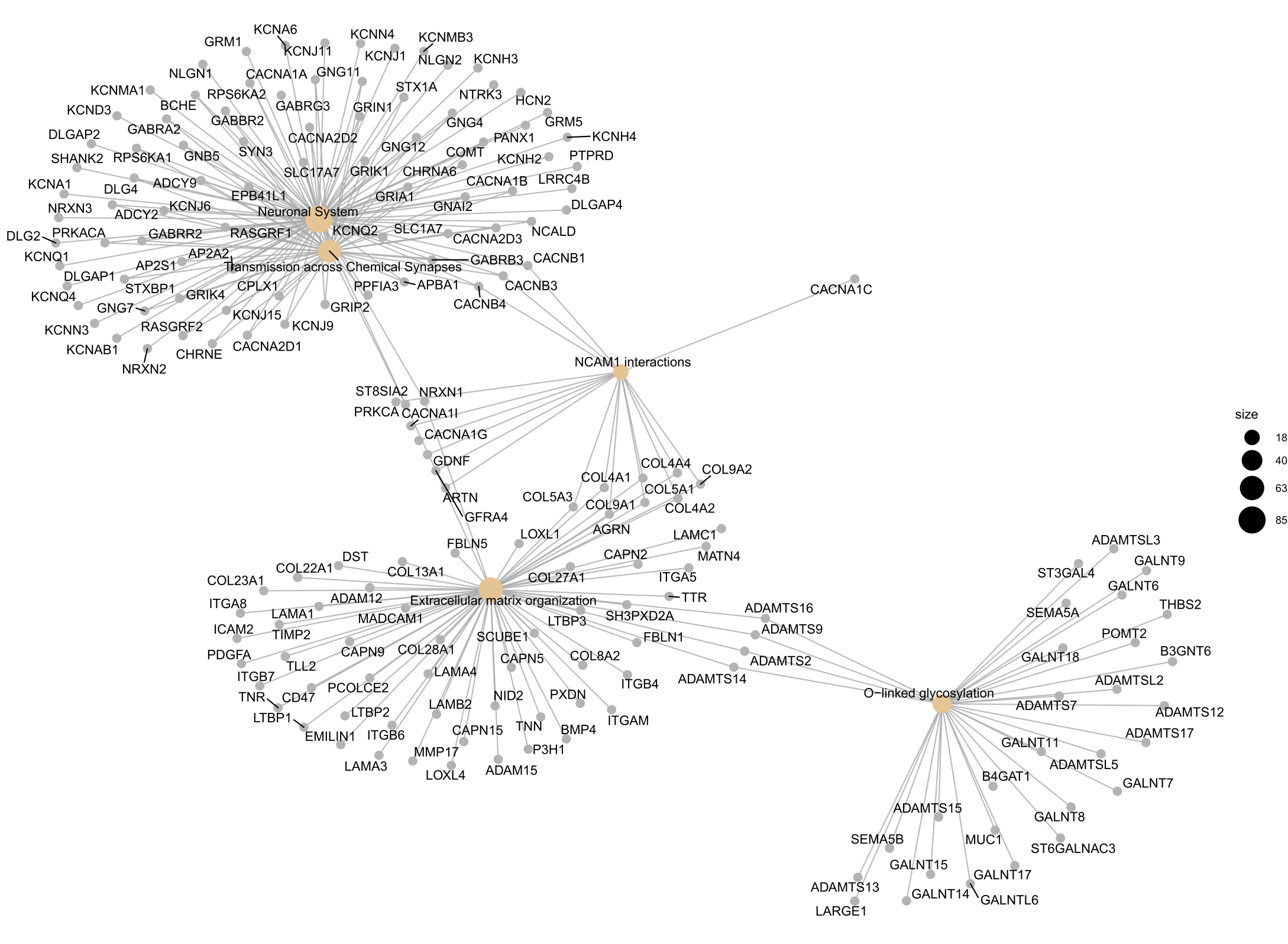
Figure S16- Interactive networks of genes from the ADNP epi-signatures
